# Supplementary material for: DNAJC9 prevents CENP-A mislocalization and chromosomal instability by maintaining the fidelity of histone supply chains
Source: EMBO J. 2024 Apr 10;43(11):5. doi: 10.1038/s44318-024-00093-6 (PMC11148058; doi:10.1038/s44318-024-00093-6)
Supplement: Supplementary file 1 — Appendix [file 44318_2024_93_MOESM1_ESM.pdf]

Appendix

DNAJC9 prevents CENP-A mislocalization and chromosomal instability by maintaining the fidelity of histone supply chains

Vinutha Balachandra, Roshan L. Shrestha[#], Colin M. Hammond[#,*], Shinjen Lin[#], Ivo A. Hendriks, Subhash Chandra Sethi, Lu Chen, Samantha Sevilla, Natasha J. Caplen, Raj Chari, Tatiana S. Karpova, Katherine McKinnon, Matthew A. M. Todd, Vishal Koparde, Ken Chih-Chien Cheng, Michael L. Nielsen, Anja Groth, and Munira A. Basrai,*

Table of contents

Page no.

Appendix Figure S1…………………..2

Appendix Figure S2…………………..3

Appendix Figure S3…………………..4

Appendix Figure S4…………………..5

Appendix Figure S5…………………..6

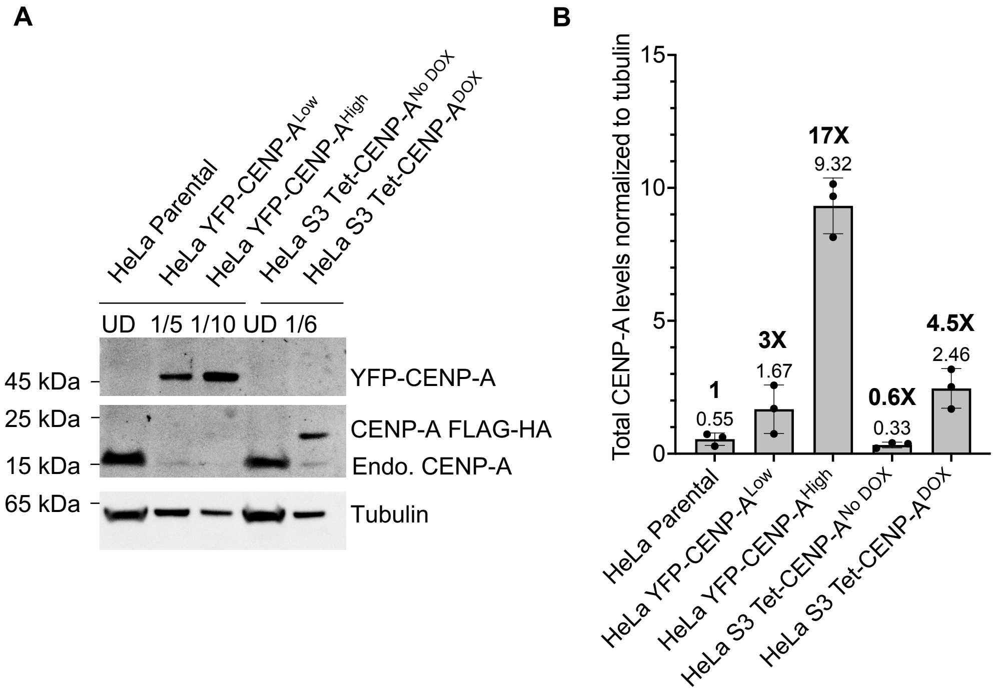

Appendix Figure S1. Western blot for expression of CENP-A in the various cell lines used in this study

A. Western blot of whole cell extracts showing CENP-A levels (YFP-tagged, FLAG-HA-tagged and endogenous) in the indicated cell lines. HeLa S3 Tet-CENP-A FLAG-HA cells were treated with 1 μg/ml DOX for 48 hrs. Diluted lysates from HeLa YFP-CENP-A^{Low} (1/5) HeLa YFP-CENP-A^{High} (1/10) and DOX-treated HeLa S3 Tet-CENP-A FLAG-HA (1/6) were loaded as shown in the figure. UD – Undiluted lysate. Blot was probed with anti-CENP-A antibody and alpha-tubulin was used as the loading control. Representative image from three biological replicates are shown.

B. Bar graphs showing total CENP-A levels (tagged and endogenous) normalized to tubulin in the indicated cell lines as described in A. Mean values, shown above the error bar, were plotted for each condition from three biological replicates. The mean value for each condition was normalized against the mean value from parental HeLa and represented as fold change values as shown in bold above the error bars. Error bars represent SD.

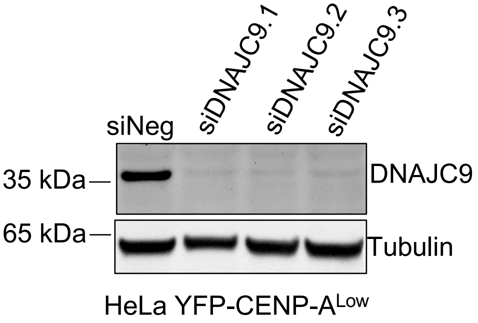

Appendix Figure S2. Depletion efficiency of DNAJC9 siRNAs

Western blot of whole cell extracts showing DNAJC9 depletion in HeLa YFP-CENP-A^{Low} cells transfected with control (siNeg) or siRNAs targeting DNAJC9 (siDNAJC9.1, siDNAJC9.2, siDNAJC9.3). All subsequent experiments described in this study were performed using siDNAJC9.3. Alpha-tubulin was used as the loading control. Representative image from three biological replicates is shown.

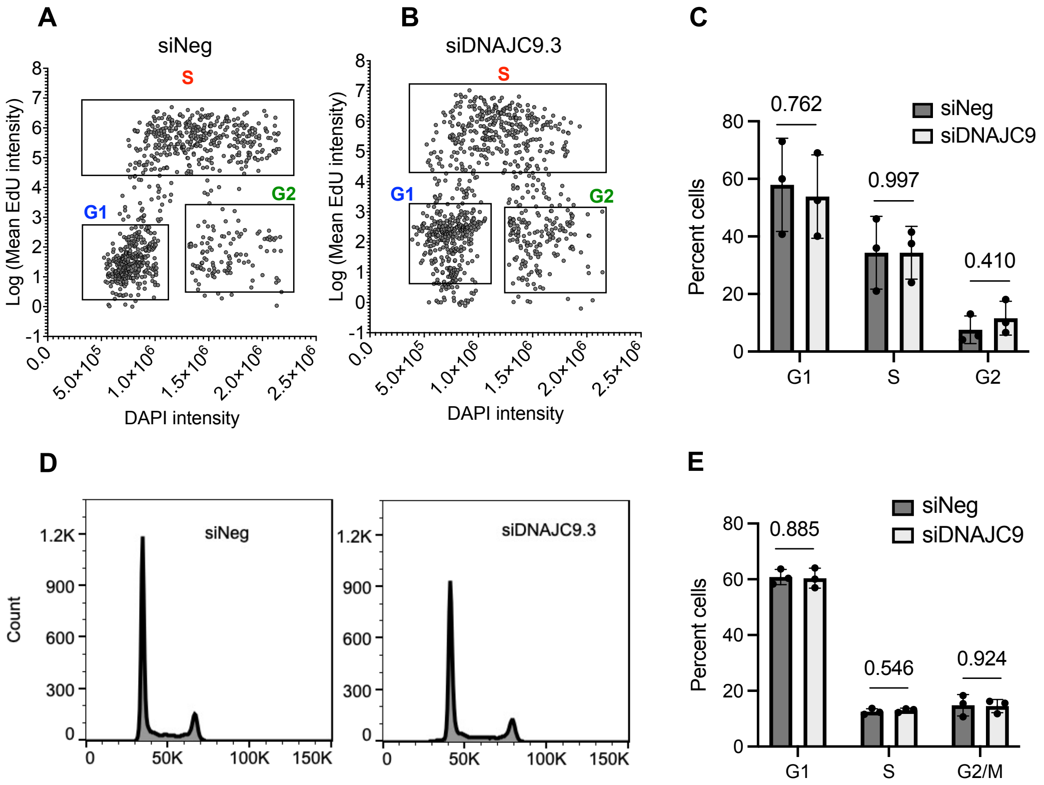

Appendix Figure S3. Cell cycle profile of DNAJC9-depleted cells.

A, B. Representative scatter plots showing DAPI intensities plotted against Log scale of mean EdU intensities in control (A) or siDNAJC9.3 (B) transfected cells. Cells were gated to G1, S or G2 stages based on EdU and DAPI intensities. The number of cells analyzed per condition from three biological replicates is shown in Fig 2D.

C. Bar charts showing proportion of G1, S and G2 cells derived from EdU-based cell cycle binning described in A and B. Statistical analysis of data from three biological replicates was performed using Unpaired t-test with Welch's correction. Mean with SD shown.

D. Flow cytometry profile of HeLa YFP-CENP-A^{Low} cells depleted with control siRNA (siNeg) or siDNAJC9.3.

E. Bar charts showing proportion of G1, S and G2 cells derived from flow cytometry analysis described in D. Statistical analysis of data from three biological replicates was performed using Unpaired t-test with Welch's correction. Mean with SD shown.

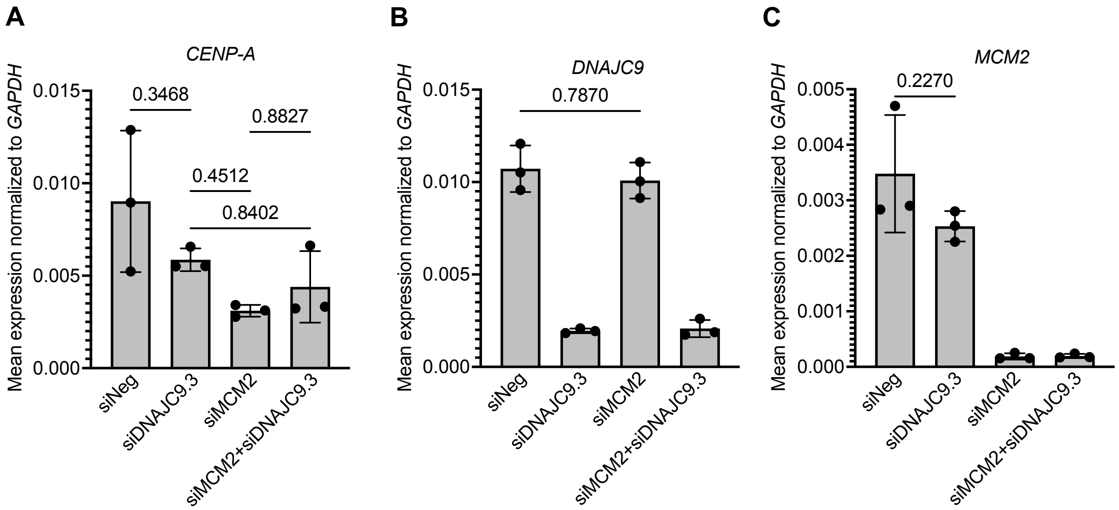

Appendix Figure S4. CENP-A mRNA levels are unaffected by depletion or co-depletion of DNAJC9 and MCM2 in HeLa YFP-CENP-A^{Low} cells

A-C. Bar graphs showing mean RNA levels of *CENP-A* (A), *DNAJC9* (B) and *MCM2* (C) normalized to *GAPDH* in control and DNAJC9-depleted cells with or without MCM2 depletion. RT-qPCR was done to analyze the RNA levels from three biological replicates. Mean values with standard deviation were plotted and p-values were calculated from one-way ANOVA with Tukey's multiple correction test.

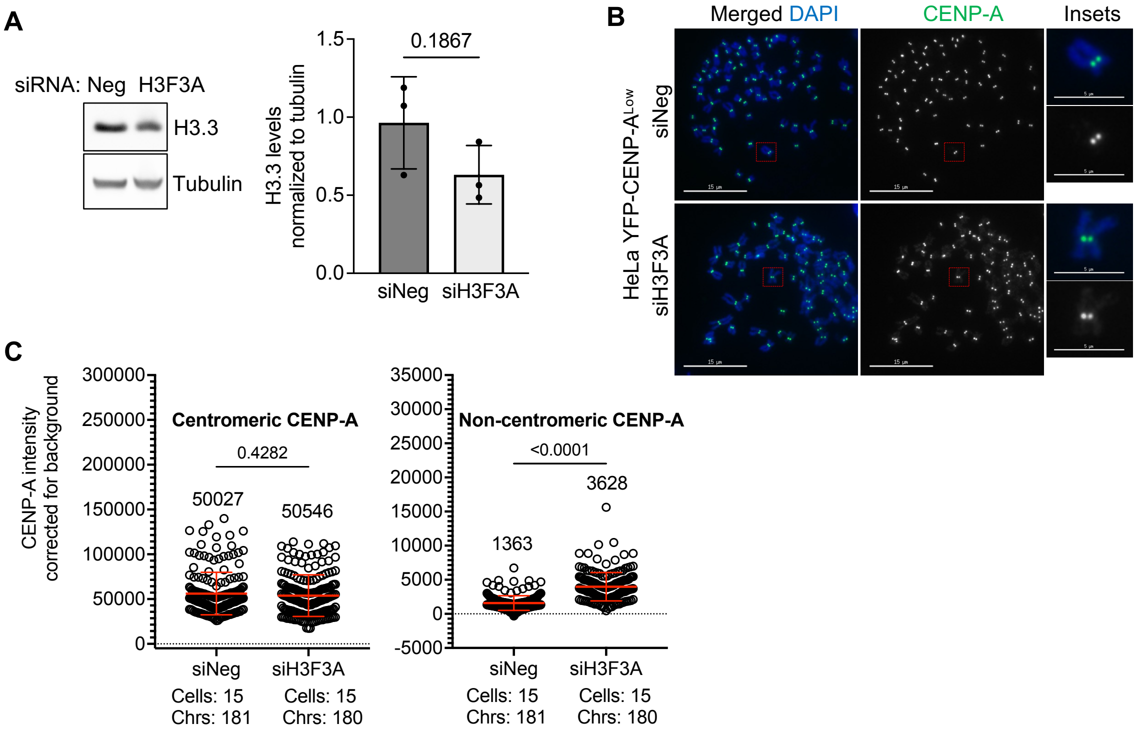

Appendix Figure S5. H3.3-depleted cells exhibit mislocalization of CENP-A to non-centromeric regions in HeLa YFP-CENP-A^{Low} cells

A. (Left panel) Western blot of whole cell extracts showing H3.3 depletion in HeLa YFP-CENP-A^{Low} cells transfected with control (siNeg) or siRNA targeting *H3F3A.* Alpha-tubulin was used as the loading control. Representative images from three biological replicates shown. (Right panel) Bar graph showing H3.3 levels normalized to tubulin as described in A. Mean with SD were plotted from three biological replicates. P-values were calculated using Unpaired t-test.

B. CENP-A is mislocalized in H3.3-depleted HeLa YFP-CENP-A^{Low} cells. Representative images of metaphase chromosome spreads immunostained for CENP-A in siNeg or siH3F3A-transfected HeLa YFP-CENP-A^{Low} cells. Scale bar–- 15 micrometer. Scale bar for insets – 5 μm.

C. Scatter plots showing CENP-A intensities corrected for background at the centromeric or non-centromeric regions as described in B, from three biological replicates. Median values are shown above graph. Each circle represents value from individual chromosome. Chrs represent the total number of chromosomes measured per condition from three independent experiments. Mean with standard deviation is shown across all measurements from three biological replicates. P-values were calculated using Mann-Whitney *U* test.
