# Supplementary figures and images for: DNAJC9 prevents CENP-A mislocalization and chromosomal instability by maintaining the fidelity of histone supply chains
Source: EMBO J. 2024 Apr 10;43(11):5. doi: 10.1038/s44318-024-00093-6 (PMC11148058; doi:10.1038/s44318-024-00093-6)

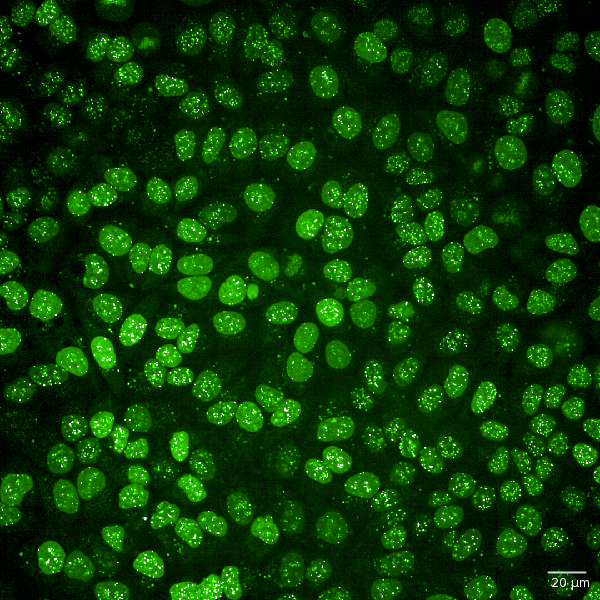

Supplement: Supplementary file 6 — Source data Fig. 1 [file 44318_2024_93_MOESM6_ESM.zip › Figure 1/1D/Primary Screen_YFP-CENPA_siDNAJC9.dib]

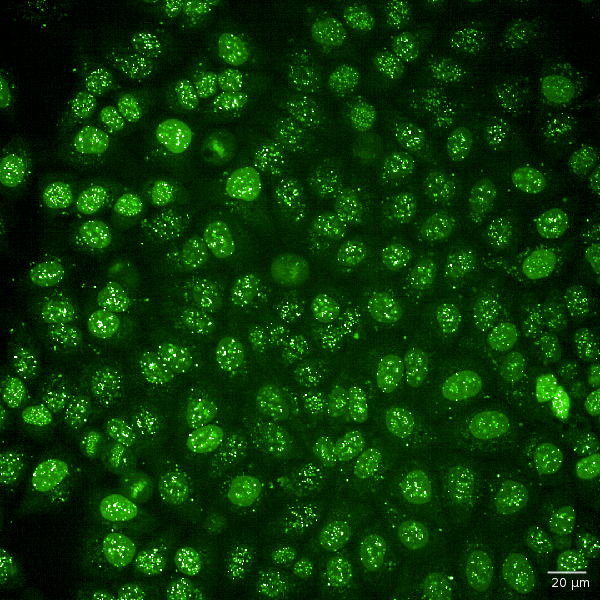

Supplement: Supplementary file 6 — Source data Fig. 1 [file 44318_2024_93_MOESM6_ESM.zip › Figure 1/1D/ Primary Screen_YFP-CENPA_siNEG.dib]

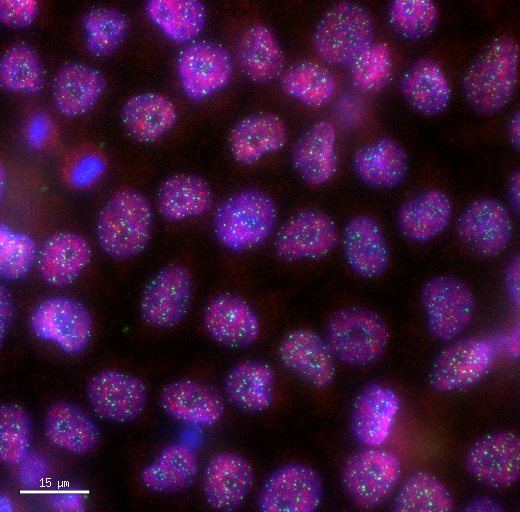

Supplement: Supplementary file 7 — Source data Fig. 2 [file 44318_2024_93_MOESM7_ESM.zip › Figure 2/2A/siNeg_IF/siNeg_30_R3D_PRJ_MERGE.tif]

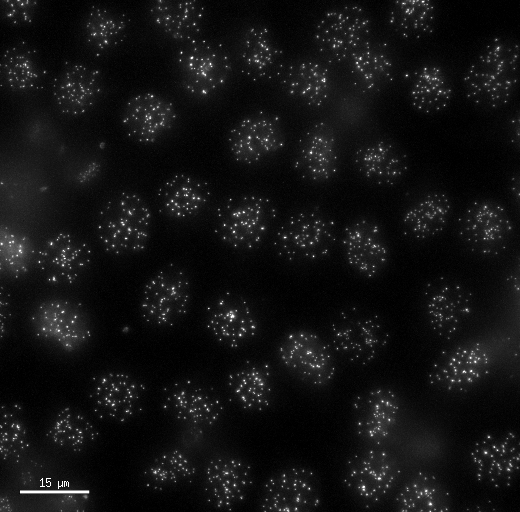

Supplement: Supplementary file 7 — Source data Fig. 2 [file 44318_2024_93_MOESM7_ESM.zip › Figure 2/2A/siNeg_IF/siNeg_30_R3D_PRJ_CENPA.tif]

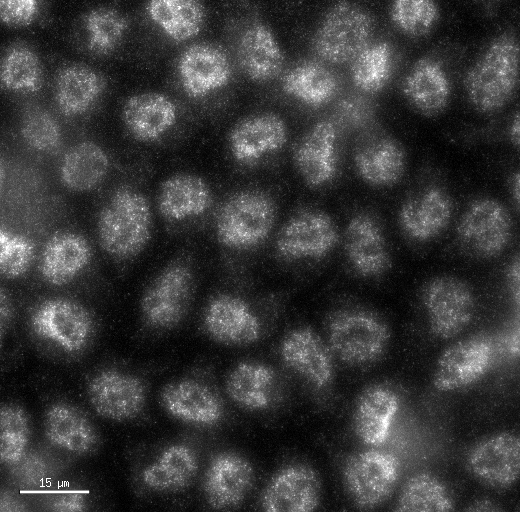

Supplement: Supplementary file 7 — Source data Fig. 2 [file 44318_2024_93_MOESM7_ESM.zip › Figure 2/2A/siNeg_IF/siNeg_30_R3D_PRJ_DNAJC9.tif]

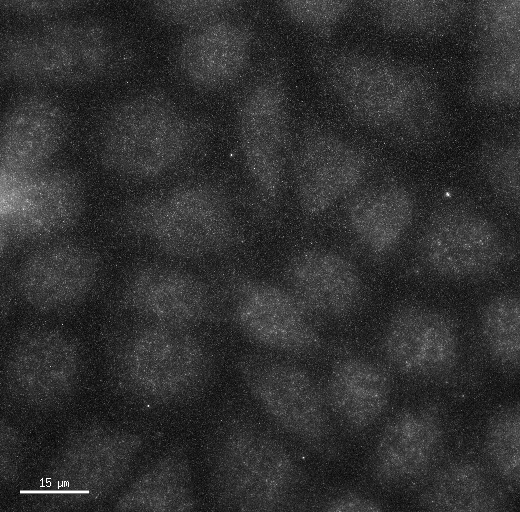

Supplement: Supplementary file 7 — Source data Fig. 2 [file 44318_2024_93_MOESM7_ESM.zip › Figure 2/2A/siDNAJC9.3_No DOX_IF/NODOX_29_R3D_PRJ_JC9.tif]

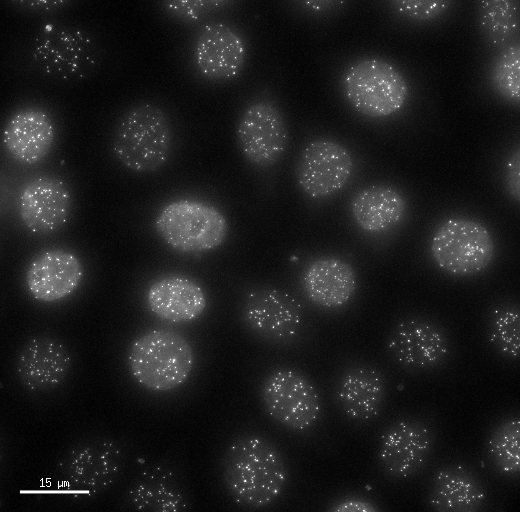

Supplement: Supplementary file 7 — Source data Fig. 2 [file 44318_2024_93_MOESM7_ESM.zip › Figure 2/2A/siDNAJC9.3_No DOX_IF/NODOX_29_R3D_PRJ_CENPA.tif]

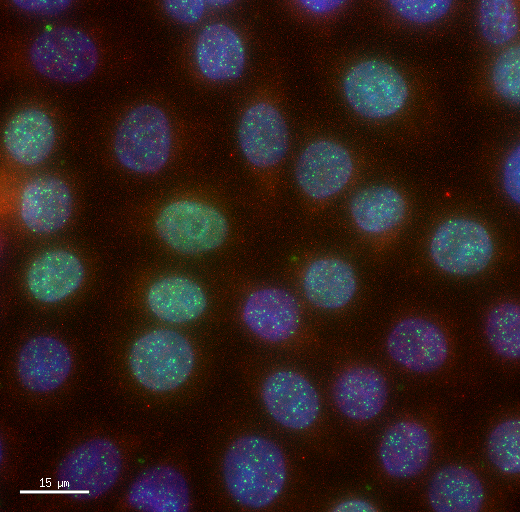

Supplement: Supplementary file 7 — Source data Fig. 2 [file 44318_2024_93_MOESM7_ESM.zip › Figure 2/2A/siDNAJC9.3_No DOX_IF/NODOX_29_R3D_PRJ_MERGE.tif]

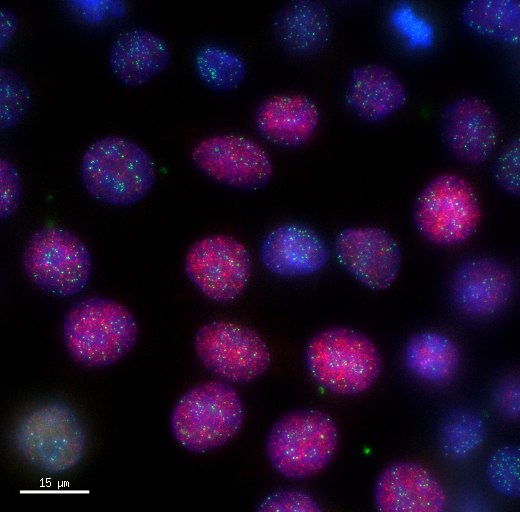

Supplement: Supplementary file 7 — Source data Fig. 2 [file 44318_2024_93_MOESM7_ESM.zip › Figure 2/2A/siDNAJC9.3_DOX_IF/DOX_21_R3D_PRJ_MERGE.tif]

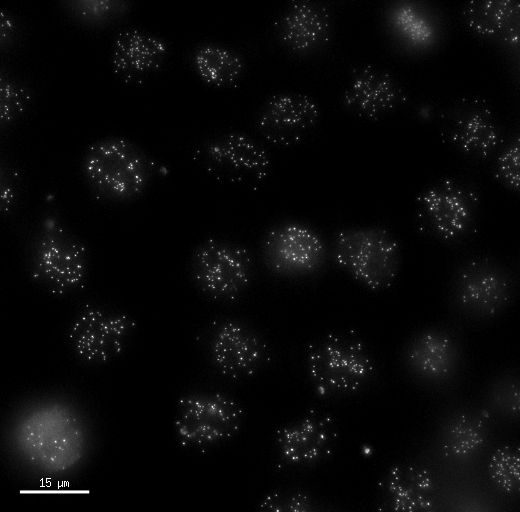

Supplement: Supplementary file 7 — Source data Fig. 2 [file 44318_2024_93_MOESM7_ESM.zip › Figure 2/2A/siDNAJC9.3_DOX_IF/DOX_21_R3D_PRJ_CENPA.tif]

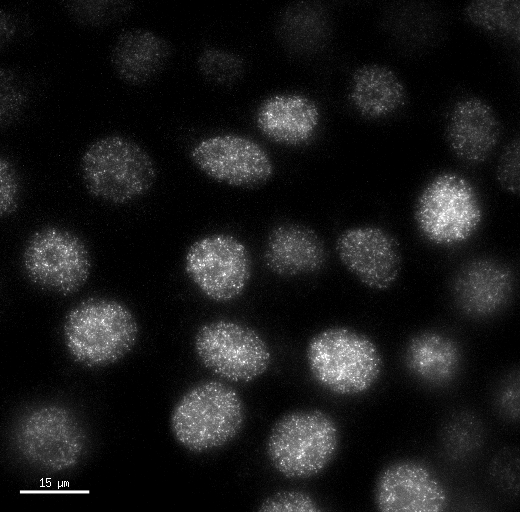

Supplement: Supplementary file 7 — Source data Fig. 2 [file 44318_2024_93_MOESM7_ESM.zip › Figure 2/2A/siDNAJC9.3_DOX_IF/DOX_21_R3D_PRJ_JC9.tif]

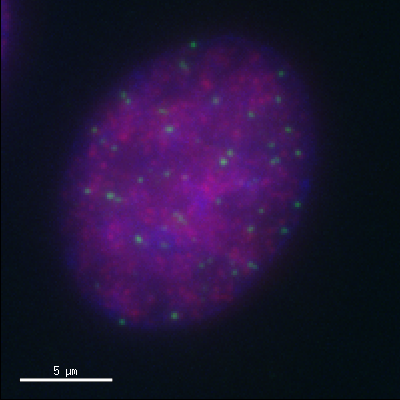

Supplement: Supplementary file 7 — Source data Fig. 2 [file 44318_2024_93_MOESM7_ESM.zip › Figure 2/2C/siNeg_IF/S_siNeg_merged.tif]

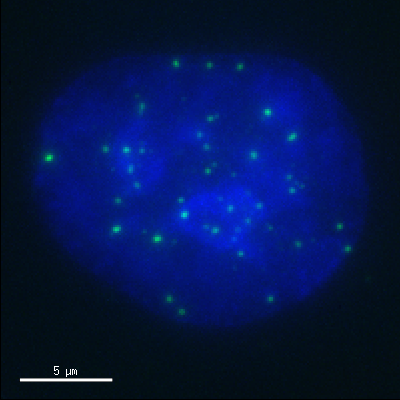

Supplement: Supplementary file 7 — Source data Fig. 2 [file 44318_2024_93_MOESM7_ESM.zip › Figure 2/2C/siNeg_IF/G2_siNeg_Merged.tif]

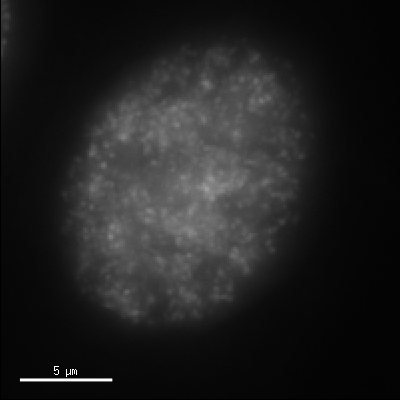

Supplement: Supplementary file 7 — Source data Fig. 2 [file 44318_2024_93_MOESM7_ESM.zip › Figure 2/2C/siNeg_IF/S_siNeg_EdU.tif]

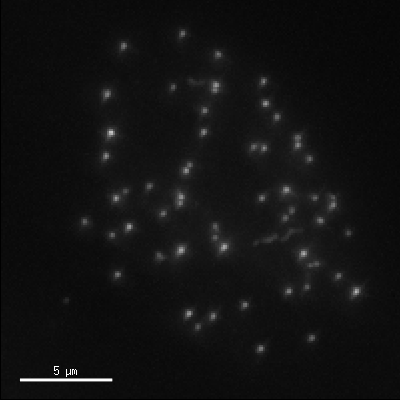

Supplement: Supplementary file 7 — Source data Fig. 2 [file 44318_2024_93_MOESM7_ESM.zip › Figure 2/2C/siNeg_IF/G1_siNeg_CENPA.tif]

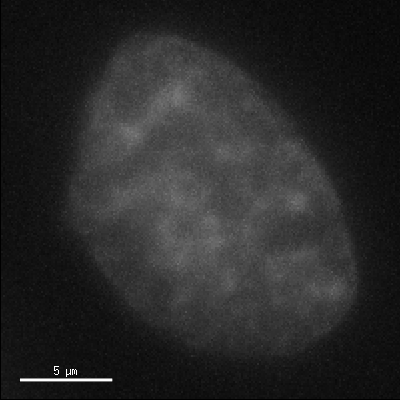

Supplement: Supplementary file 7 — Source data Fig. 2 [file 44318_2024_93_MOESM7_ESM.zip › Figure 2/2C/siNeg_IF/G1_siNeg_DAPI.tif]

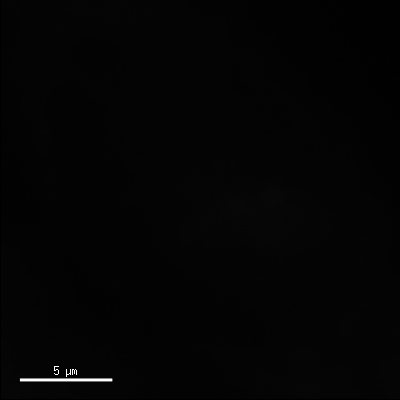

Supplement: Supplementary file 7 — Source data Fig. 2 [file 44318_2024_93_MOESM7_ESM.zip › Figure 2/2C/siNeg_IF/G1_siNeg_EdU.tif]

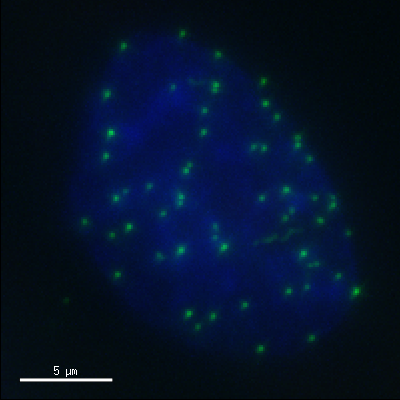

Supplement: Supplementary file 7 — Source data Fig. 2 [file 44318_2024_93_MOESM7_ESM.zip › Figure 2/2C/siNeg_IF/G1_siNeg_merged.tif]

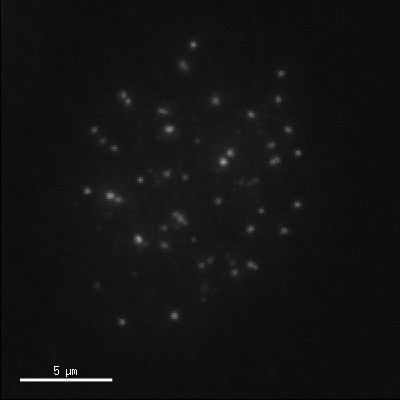

Supplement: Supplementary file 7 — Source data Fig. 2 [file 44318_2024_93_MOESM7_ESM.zip › Figure 2/2C/siNeg_IF/S_siNeg_CENPA.tif]

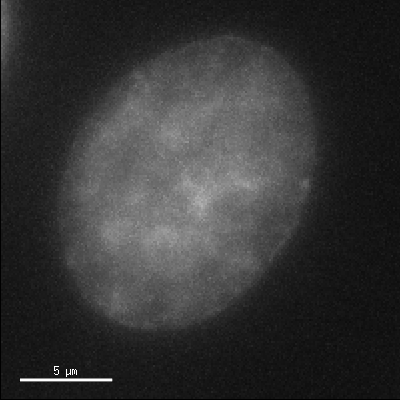

Supplement: Supplementary file 7 — Source data Fig. 2 [file 44318_2024_93_MOESM7_ESM.zip › Figure 2/2C/siNeg_IF/S_siNeg_DAPI.tif]

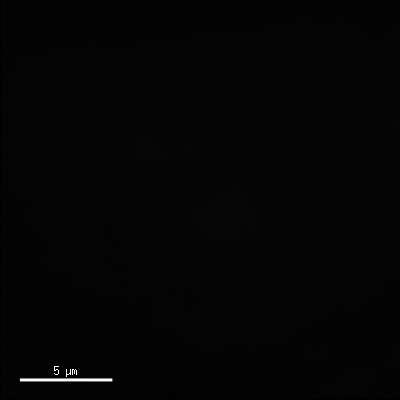

Supplement: Supplementary file 7 — Source data Fig. 2 [file 44318_2024_93_MOESM7_ESM.zip › Figure 2/2C/siNeg_IF/G2_siNeg_Edu.tif]

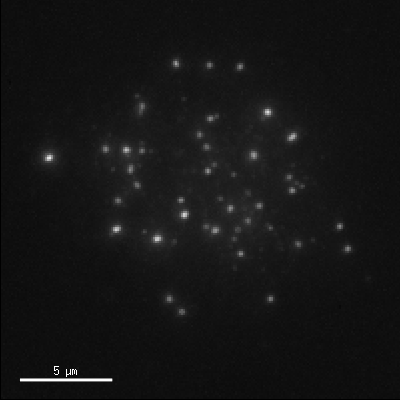

Supplement: Supplementary file 7 — Source data Fig. 2 [file 44318_2024_93_MOESM7_ESM.zip › Figure 2/2C/siNeg_IF/G2_siNeg_CENPA.tif]

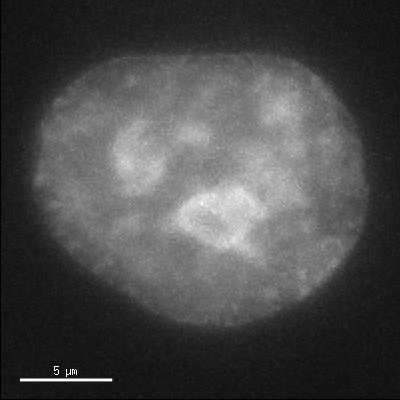

Supplement: Supplementary file 7 — Source data Fig. 2 [file 44318_2024_93_MOESM7_ESM.zip › Figure 2/2C/siNeg_IF/G2_siNeg_DAPI.tif]

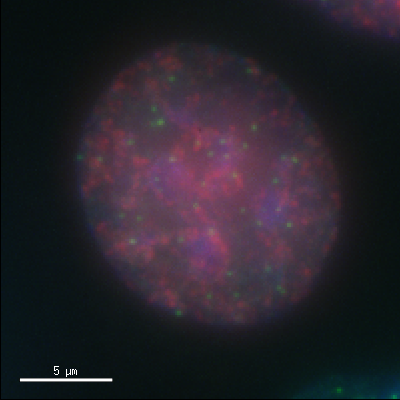

Supplement: Supplementary file 7 — Source data Fig. 2 [file 44318_2024_93_MOESM7_ESM.zip › Figure 2/2C/siDNAJC9.3_IF/S_siDNAJC9.3_Merged.tif]

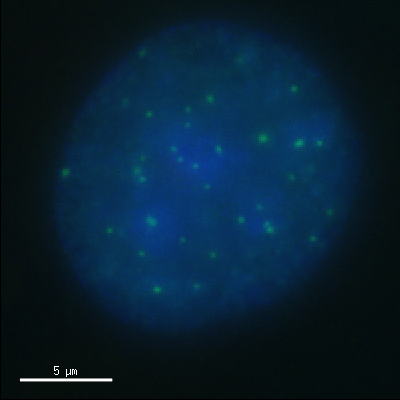

Supplement: Supplementary file 7 — Source data Fig. 2 [file 44318_2024_93_MOESM7_ESM.zip › Figure 2/2C/siDNAJC9.3_IF/G2_siDNAJC9.3_Merged.tif]

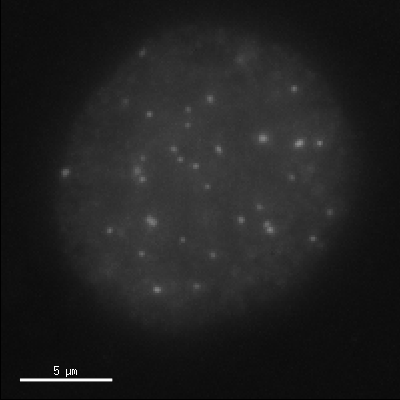

Supplement: Supplementary file 7 — Source data Fig. 2 [file 44318_2024_93_MOESM7_ESM.zip › Figure 2/2C/siDNAJC9.3_IF/G2_siDNAJC9.3_CENPA.tif]

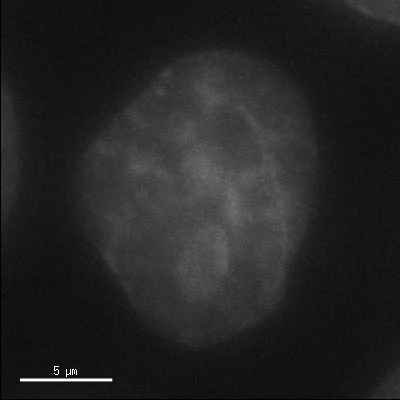

Supplement: Supplementary file 7 — Source data Fig. 2 [file 44318_2024_93_MOESM7_ESM.zip › Figure 2/2C/siDNAJC9.3_IF/G1_siDNAJC9.3_DAPI.tif]

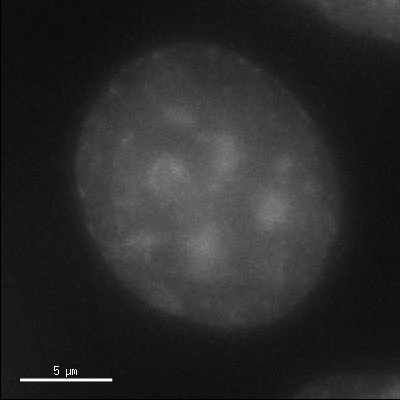

Supplement: Supplementary file 7 — Source data Fig. 2 [file 44318_2024_93_MOESM7_ESM.zip › Figure 2/2C/siDNAJC9.3_IF/S_siDNAJC9.3_DAPI.tif]

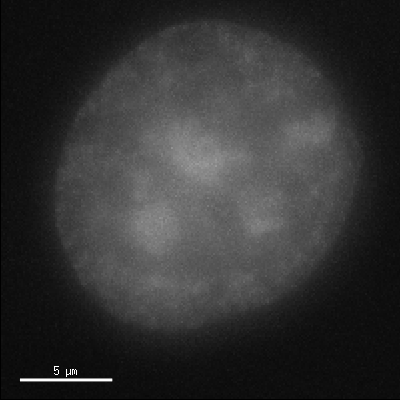

Supplement: Supplementary file 7 — Source data Fig. 2 [file 44318_2024_93_MOESM7_ESM.zip › Figure 2/2C/siDNAJC9.3_IF/G2_siDNAJC9.3_DAPI.tif]

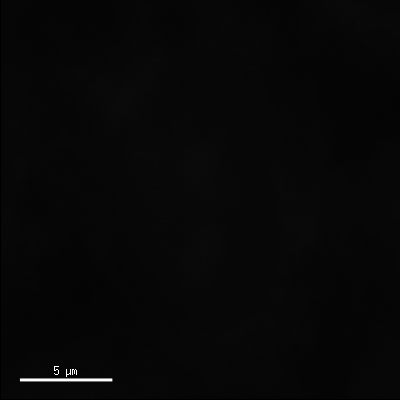

Supplement: Supplementary file 7 — Source data Fig. 2 [file 44318_2024_93_MOESM7_ESM.zip › Figure 2/2C/siDNAJC9.3_IF/G1_siDNAJC9.3_EdU.tif]

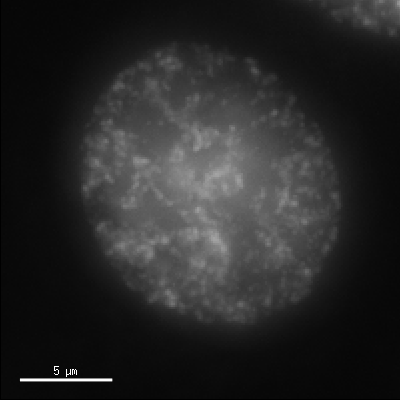

Supplement: Supplementary file 7 — Source data Fig. 2 [file 44318_2024_93_MOESM7_ESM.zip › Figure 2/2C/siDNAJC9.3_IF/S_siDNAJC9.3_EdU.tif]

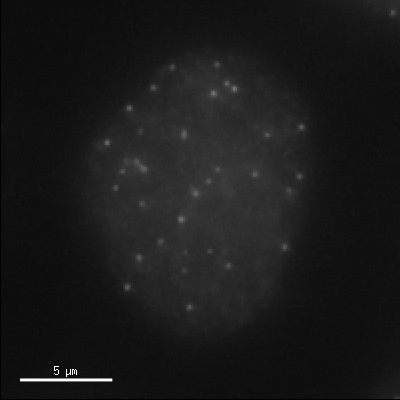

Supplement: Supplementary file 7 — Source data Fig. 2 [file 44318_2024_93_MOESM7_ESM.zip › Figure 2/2C/siDNAJC9.3_IF/G1_siDNAJC9.3_CENPA.tif]

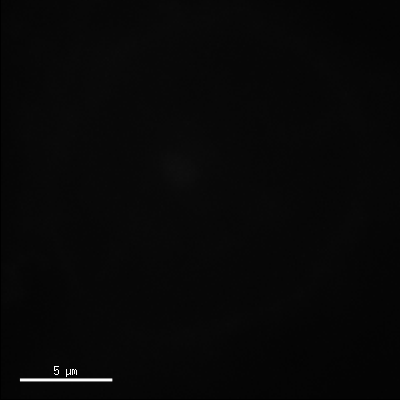

Supplement: Supplementary file 7 — Source data Fig. 2 [file 44318_2024_93_MOESM7_ESM.zip › Figure 2/2C/siDNAJC9.3_IF/G2_siDNAJC9.3_EdU.tif]

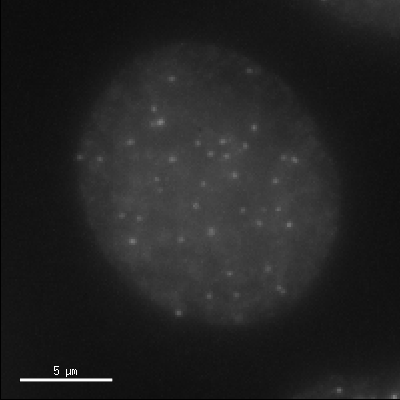

Supplement: Supplementary file 7 — Source data Fig. 2 [file 44318_2024_93_MOESM7_ESM.zip › Figure 2/2C/siDNAJC9.3_IF/S_siDNAJC9.3_CENPA.tif]

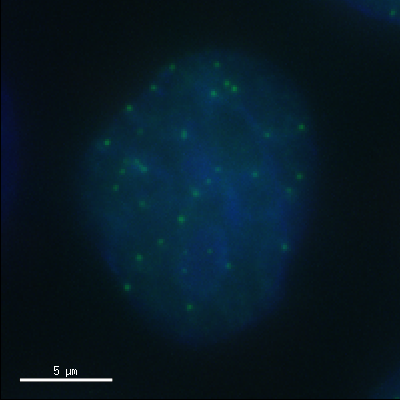

Supplement: Supplementary file 7 — Source data Fig. 2 [file 44318_2024_93_MOESM7_ESM.zip › Figure 2/2C/siDNAJC9.3_IF/G1_siDNAJC9.3_Merged.tif]

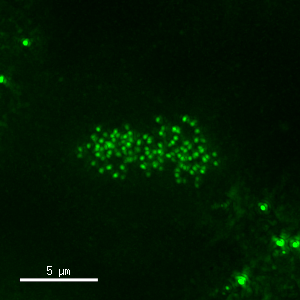

Supplement: Supplementary file 8 — Source data Fig. 3 [file 44318_2024_93_MOESM8_ESM.zip › Figure 3/3E/siNeg_CENPA.tif]

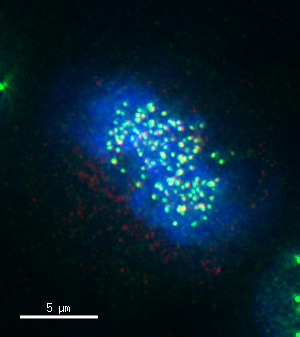

Supplement: Supplementary file 8 — Source data Fig. 3 [file 44318_2024_93_MOESM8_ESM.zip › Figure 3/3E/siDNAJC9_Merged.tif]

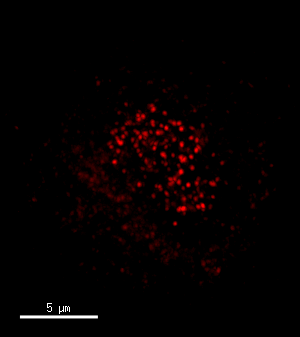

Supplement: Supplementary file 8 — Source data Fig. 3 [file 44318_2024_93_MOESM8_ESM.zip › Figure 3/3E/siDNAJC9_NUF2.tif]

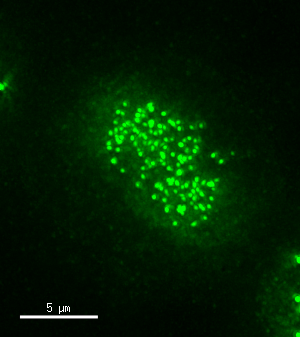

Supplement: Supplementary file 8 — Source data Fig. 3 [file 44318_2024_93_MOESM8_ESM.zip › Figure 3/3E/siDNAJC9_CENPA.tif]

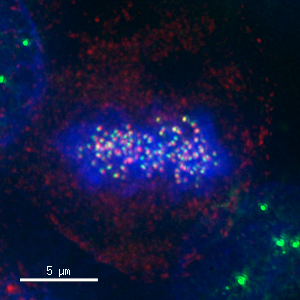

Supplement: Supplementary file 8 — Source data Fig. 3 [file 44318_2024_93_MOESM8_ESM.zip › Figure 3/3E/siNeg_Merged.tif]

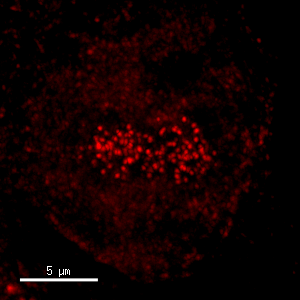

Supplement: Supplementary file 8 — Source data Fig. 3 [file 44318_2024_93_MOESM8_ESM.zip › Figure 3/3E/siNeg_NUF2.tif]

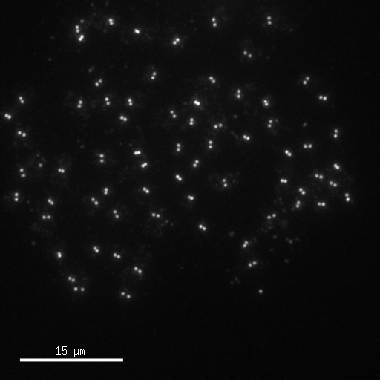

Supplement: Supplementary file 8 — Source data Fig. 3 [file 44318_2024_93_MOESM8_ESM.zip › Figure 3/3C/siNeg_CENPC.tif]

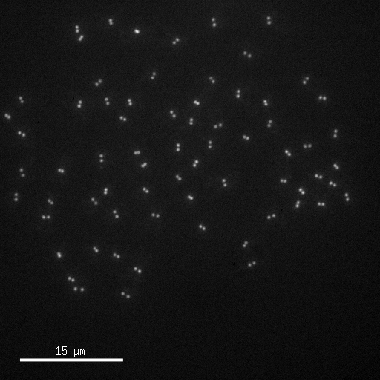

Supplement: Supplementary file 8 — Source data Fig. 3 [file 44318_2024_93_MOESM8_ESM.zip › Figure 3/3C/siNeg_CENPA.tif]

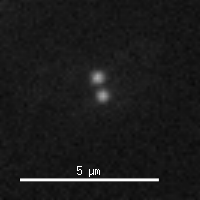

Supplement: Supplementary file 8 — Source data Fig. 3 [file 44318_2024_93_MOESM8_ESM.zip › Figure 3/3C/siNeg_CENPA_INSET.tif]

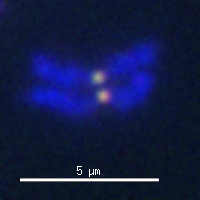

Supplement: Supplementary file 8 — Source data Fig. 3 [file 44318_2024_93_MOESM8_ESM.zip › Figure 3/3C/siNeg_Merged_INSET.tif]

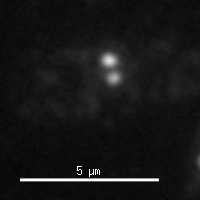

Supplement: Supplementary file 8 — Source data Fig. 3 [file 44318_2024_93_MOESM8_ESM.zip › Figure 3/3C/siDNAJC9_CENPC_INSET.tif]

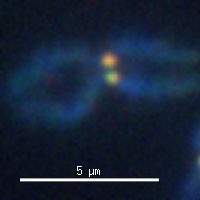

Supplement: Supplementary file 8 — Source data Fig. 3 [file 44318_2024_93_MOESM8_ESM.zip › Figure 3/3C/siDNAJC9_Merged_INSET.tif]

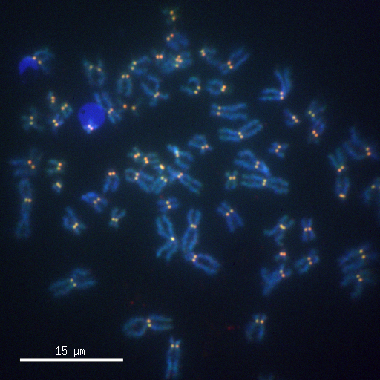

Supplement: Supplementary file 8 — Source data Fig. 3 [file 44318_2024_93_MOESM8_ESM.zip › Figure 3/3C/siDNAJC9_Merged.tif]

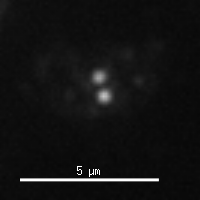

Supplement: Supplementary file 8 — Source data Fig. 3 [file 44318_2024_93_MOESM8_ESM.zip › Figure 3/3C/siNeg_CENPC_INSET.tif]

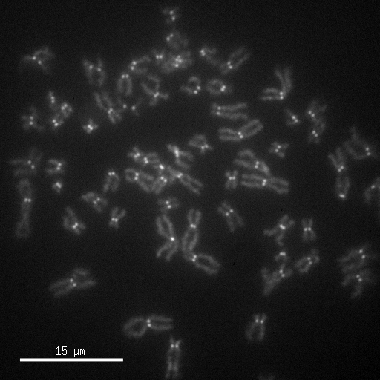

Supplement: Supplementary file 8 — Source data Fig. 3 [file 44318_2024_93_MOESM8_ESM.zip › Figure 3/3C/siDNAJC9_CENPA.tif]

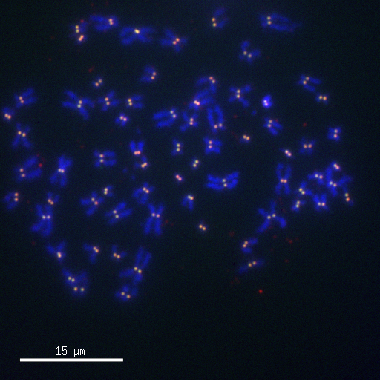

Supplement: Supplementary file 8 — Source data Fig. 3 [file 44318_2024_93_MOESM8_ESM.zip › Figure 3/3C/siNeg_Merged.tif]

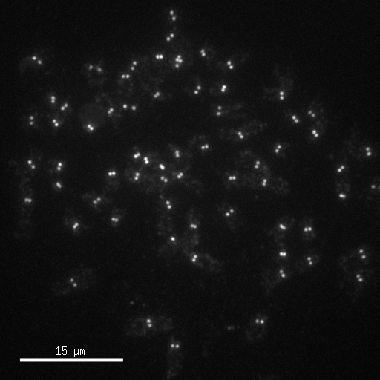

Supplement: Supplementary file 8 — Source data Fig. 3 [file 44318_2024_93_MOESM8_ESM.zip › Figure 3/3C/siDNAJC9_CENPC.tif]

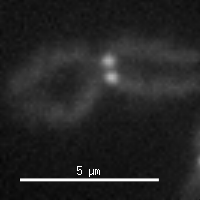

Supplement: Supplementary file 8 — Source data Fig. 3 [file 44318_2024_93_MOESM8_ESM.zip › Figure 3/3C/siDNAJC9_CENPA_INSET.tif]

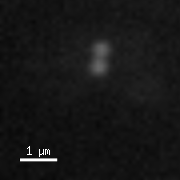

Supplement: Supplementary file 8 — Source data Fig. 3 [file 44318_2024_93_MOESM8_ESM.zip › Figure 3/3A/siNegative_CENPA_INSET.tif]

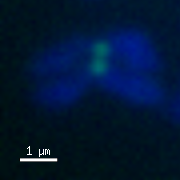

Supplement: Supplementary file 8 — Source data Fig. 3 [file 44318_2024_93_MOESM8_ESM.zip › Figure 3/3A/siNegative_Merged_INSET.tif]

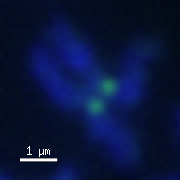

Supplement: Supplementary file 8 — Source data Fig. 3 [file 44318_2024_93_MOESM8_ESM.zip › Figure 3/3A/siDNAJC9_Merged_INSET.tif]

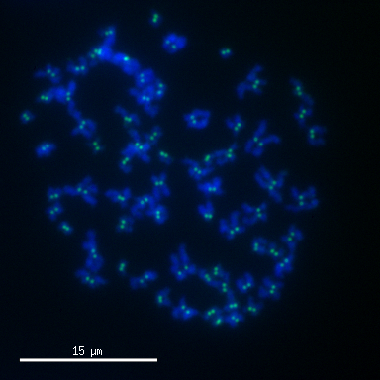

Supplement: Supplementary file 8 — Source data Fig. 3 [file 44318_2024_93_MOESM8_ESM.zip › Figure 3/3A/siDNAJC9_Merged.tif]

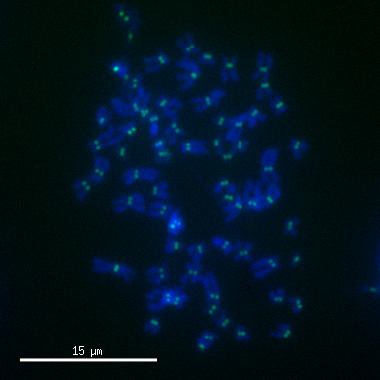

Supplement: Supplementary file 8 — Source data Fig. 3 [file 44318_2024_93_MOESM8_ESM.zip › Figure 3/3A/siNegative_Merged.tif]

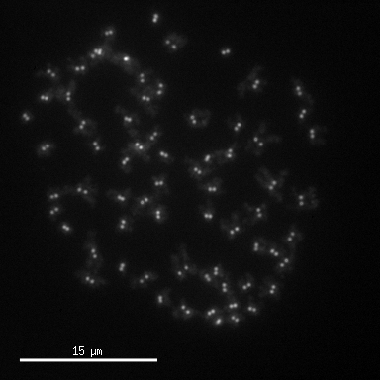

Supplement: Supplementary file 8 — Source data Fig. 3 [file 44318_2024_93_MOESM8_ESM.zip › Figure 3/3A/siDNAJC9_CENPA.tif]

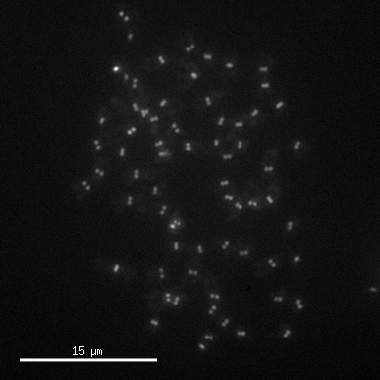

Supplement: Supplementary file 8 — Source data Fig. 3 [file 44318_2024_93_MOESM8_ESM.zip › Figure 3/3A/siNegative_CENPA.tif]

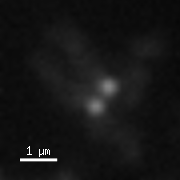

Supplement: Supplementary file 8 — Source data Fig. 3 [file 44318_2024_93_MOESM8_ESM.zip › Figure 3/3A/siDNAJC9_CENPA_INSET.tif]

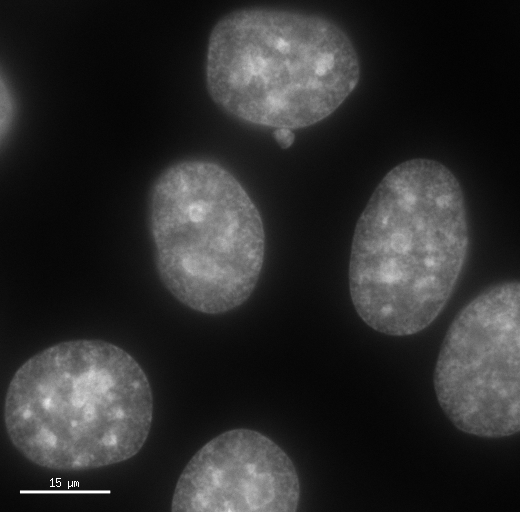

Supplement: Supplementary file 8 — Source data Fig. 3 [file 44318_2024_93_MOESM8_ESM.zip › Figure 3/3F/Representative_Micronuclei.tif]

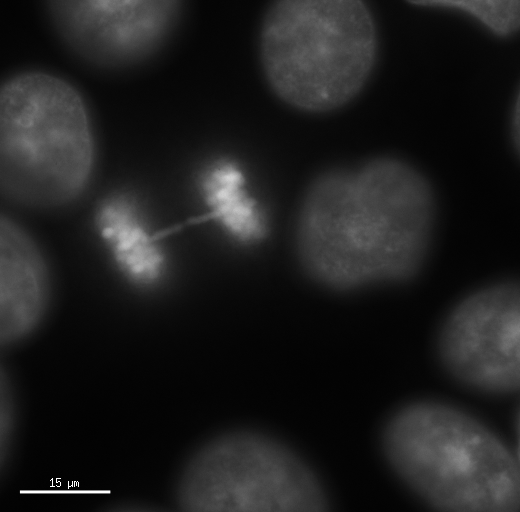

Supplement: Supplementary file 8 — Source data Fig. 3 [file 44318_2024_93_MOESM8_ESM.zip › Figure 3/3G/Representative_Segregation_defect_2.tif]

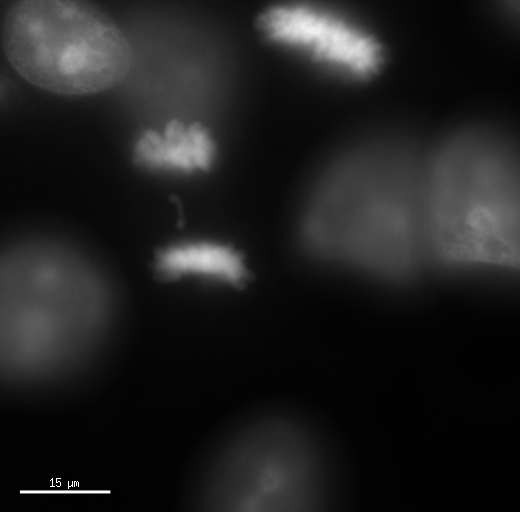

Supplement: Supplementary file 8 — Source data Fig. 3 [file 44318_2024_93_MOESM8_ESM.zip › Figure 3/3G/Representative_Segregation_defect_1.tif]

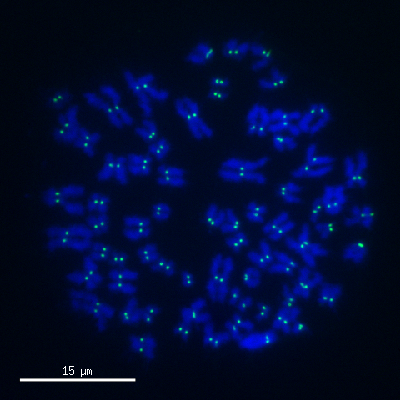

Supplement: Supplementary file 11 — Source data Fig. 6 [file 44318_2024_93_MOESM11_ESM.zip › Figure 6/6B/siNeg/Merged.tif]

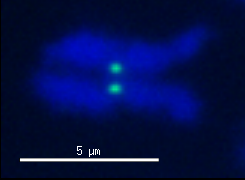

Supplement: Supplementary file 11 — Source data Fig. 6 [file 44318_2024_93_MOESM11_ESM.zip › Figure 6/6B/siNeg/Merged_INSET.tif]

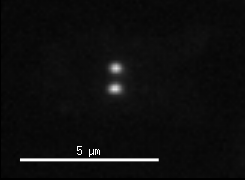

Supplement: Supplementary file 11 — Source data Fig. 6 [file 44318_2024_93_MOESM11_ESM.zip › Figure 6/6B/siNeg/CENPA_INSET.tif]

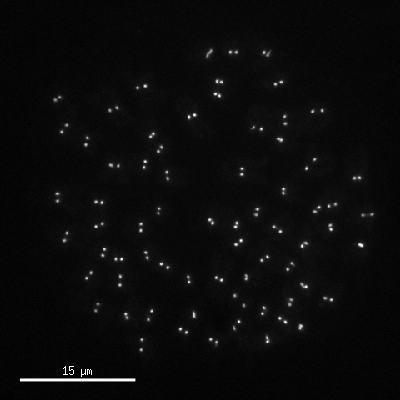

Supplement: Supplementary file 11 — Source data Fig. 6 [file 44318_2024_93_MOESM11_ESM.zip › Figure 6/6B/siNeg/CENPA.tif]

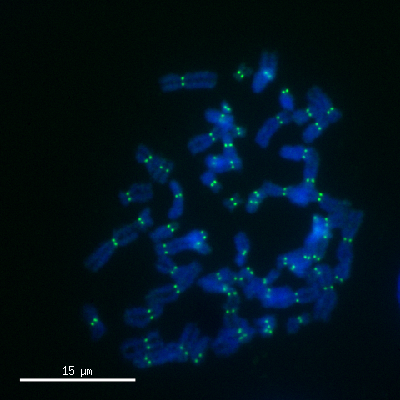

Supplement: Supplementary file 11 — Source data Fig. 6 [file 44318_2024_93_MOESM11_ESM.zip › Figure 6/6B/siDNAJC9.3/Merged.tif]

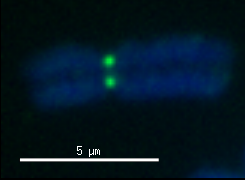

Supplement: Supplementary file 11 — Source data Fig. 6 [file 44318_2024_93_MOESM11_ESM.zip › Figure 6/6B/siDNAJC9.3/Merged_INSET.tif]

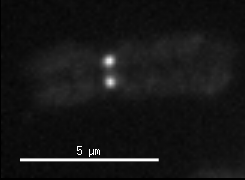

Supplement: Supplementary file 11 — Source data Fig. 6 [file 44318_2024_93_MOESM11_ESM.zip › Figure 6/6B/siDNAJC9.3/CENPA_INSET.tif]

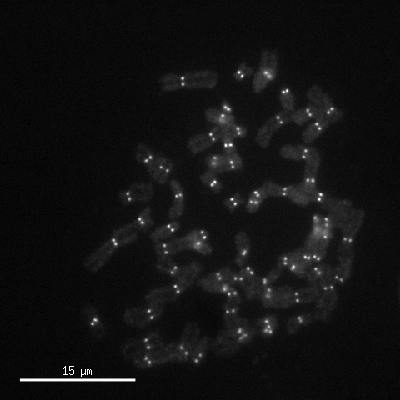

Supplement: Supplementary file 11 — Source data Fig. 6 [file 44318_2024_93_MOESM11_ESM.zip › Figure 6/6B/siDNAJC9.3/CENPA.tif]

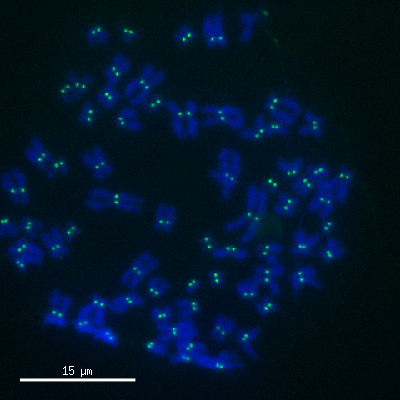

Supplement: Supplementary file 11 — Source data Fig. 6 [file 44318_2024_93_MOESM11_ESM.zip › Figure 6/6B/siMCM2/Merged.tif]

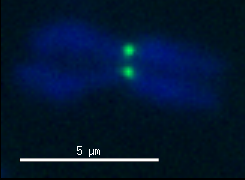

Supplement: Supplementary file 11 — Source data Fig. 6 [file 44318_2024_93_MOESM11_ESM.zip › Figure 6/6B/siMCM2/Merged_INSET.tif]

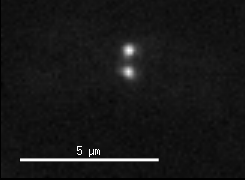

Supplement: Supplementary file 11 — Source data Fig. 6 [file 44318_2024_93_MOESM11_ESM.zip › Figure 6/6B/siMCM2/CENPA_INSET.tif]

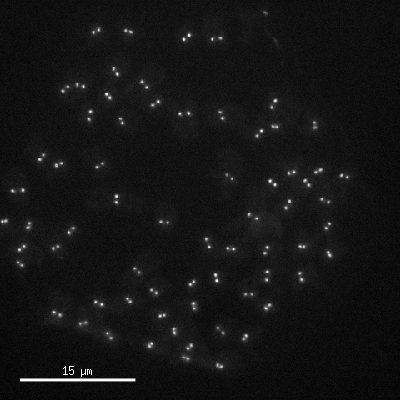

Supplement: Supplementary file 11 — Source data Fig. 6 [file 44318_2024_93_MOESM11_ESM.zip › Figure 6/6B/siMCM2/CENPA.tif]

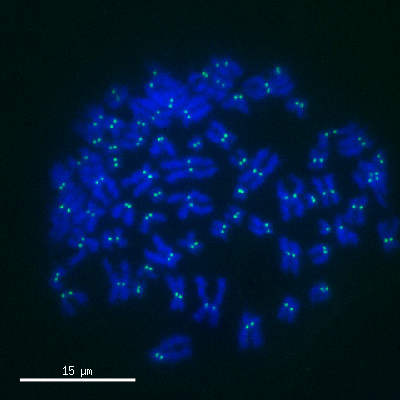

Supplement: Supplementary file 11 — Source data Fig. 6 [file 44318_2024_93_MOESM11_ESM.zip › Figure 6/6B/siMCM2+siDNAJC9.3/Merged.tif]

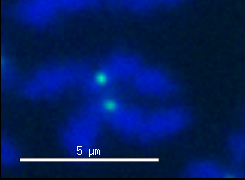

Supplement: Supplementary file 11 — Source data Fig. 6 [file 44318_2024_93_MOESM11_ESM.zip › Figure 6/6B/siMCM2+siDNAJC9.3/Merged_INSET.tif]

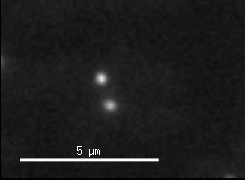

Supplement: Supplementary file 11 — Source data Fig. 6 [file 44318_2024_93_MOESM11_ESM.zip › Figure 6/6B/siMCM2+siDNAJC9.3/CENPA_INSET.tif]

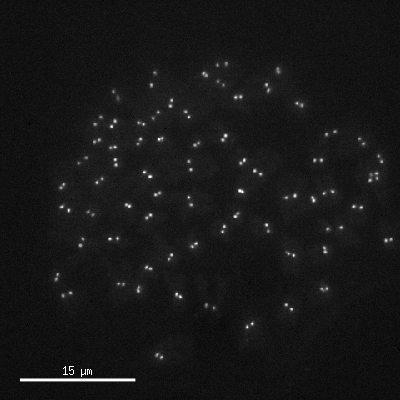

Supplement: Supplementary file 11 — Source data Fig. 6 [file 44318_2024_93_MOESM11_ESM.zip › Figure 6/6B/siMCM2+siDNAJC9.3/CENPA.tif]

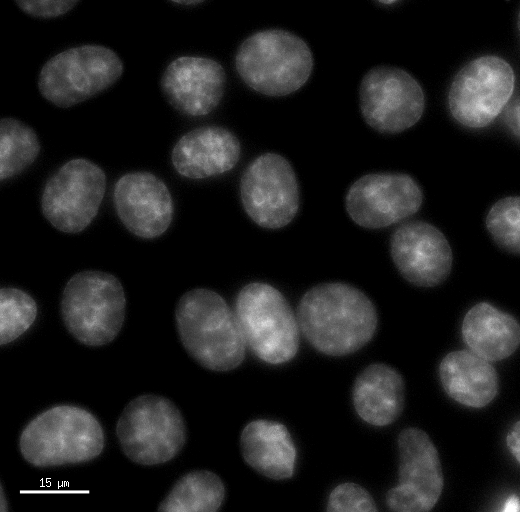

Supplement: Supplementary file 12 — Source data Fig. 7 [file 44318_2024_93_MOESM12_ESM.zip › Figure 7/7B/No vector /DAPI.tif]

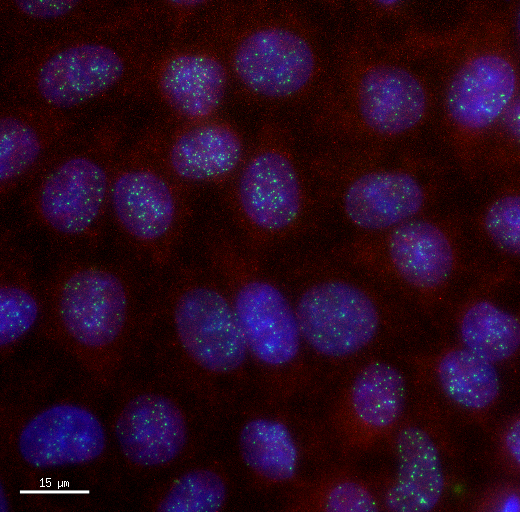

Supplement: Supplementary file 12 — Source data Fig. 7 [file 44318_2024_93_MOESM12_ESM.zip › Figure 7/7B/No vector /Merged.tif]

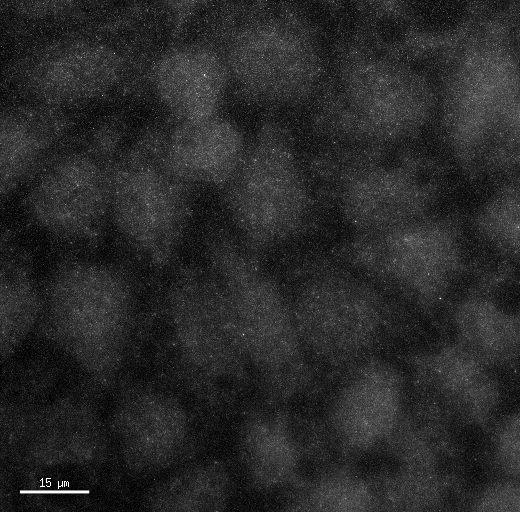

Supplement: Supplementary file 12 — Source data Fig. 7 [file 44318_2024_93_MOESM12_ESM.zip › Figure 7/7B/No vector /FLAG-DNAJC9.tif]

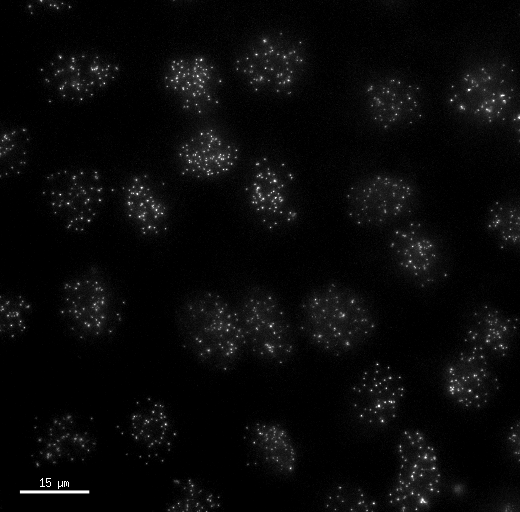

Supplement: Supplementary file 12 — Source data Fig. 7 [file 44318_2024_93_MOESM12_ESM.zip › Figure 7/7B/No vector /CENPA.tif]

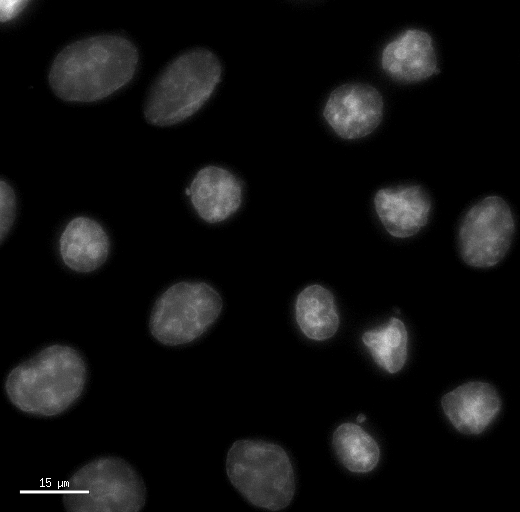

Supplement: Supplementary file 12 — Source data Fig. 7 [file 44318_2024_93_MOESM12_ESM.zip › Figure 7/7B/J/DAPI.tif]

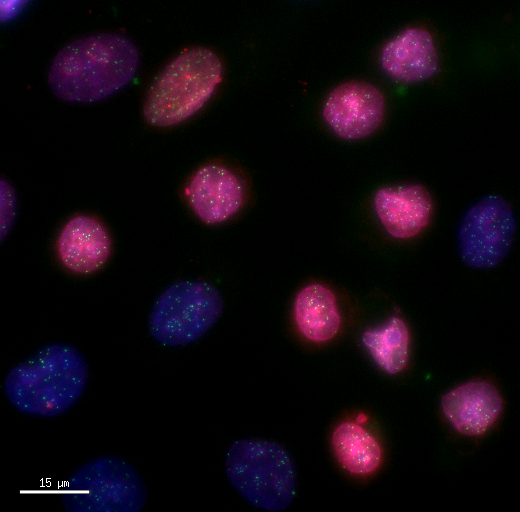

Supplement: Supplementary file 12 — Source data Fig. 7 [file 44318_2024_93_MOESM12_ESM.zip › Figure 7/7B/J/Merged.tif]

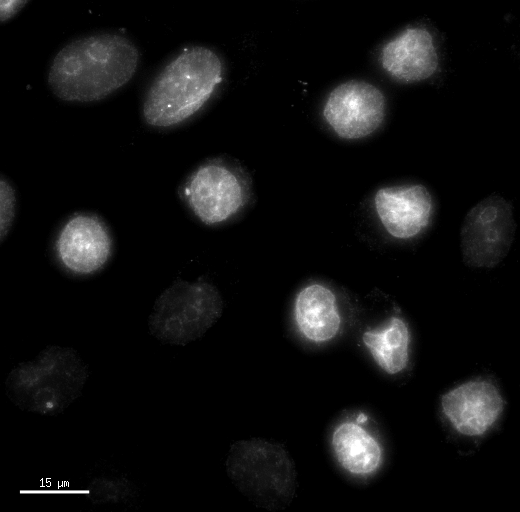

Supplement: Supplementary file 12 — Source data Fig. 7 [file 44318_2024_93_MOESM12_ESM.zip › Figure 7/7B/J/FLAG-DNAJC9.tif]

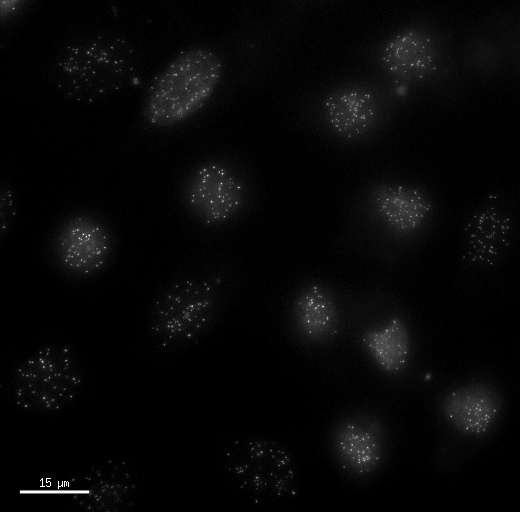

Supplement: Supplementary file 12 — Source data Fig. 7 [file 44318_2024_93_MOESM12_ESM.zip › Figure 7/7B/J/CENPA.tif]

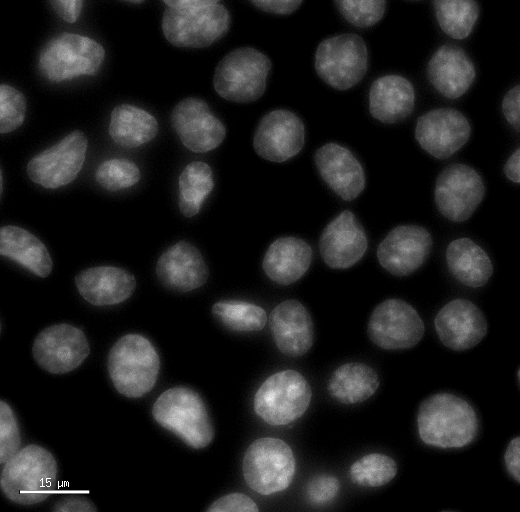

Supplement: Supplementary file 12 — Source data Fig. 7 [file 44318_2024_93_MOESM12_ESM.zip › Figure 7/7B/4A/DAPI.tif]

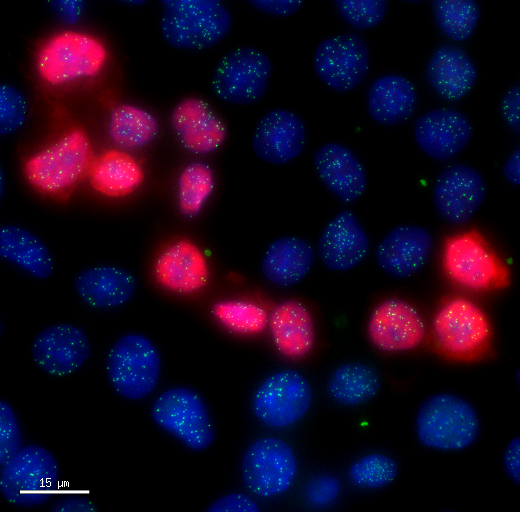

Supplement: Supplementary file 12 — Source data Fig. 7 [file 44318_2024_93_MOESM12_ESM.zip › Figure 7/7B/4A/Merged.tif]

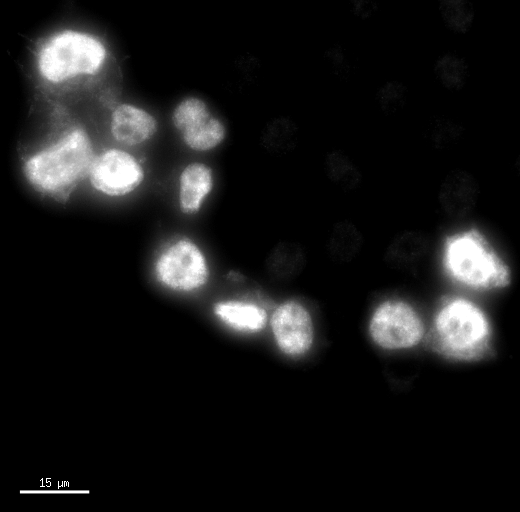

Supplement: Supplementary file 12 — Source data Fig. 7 [file 44318_2024_93_MOESM12_ESM.zip › Figure 7/7B/4A/FLAG-DNAJC9.tif]

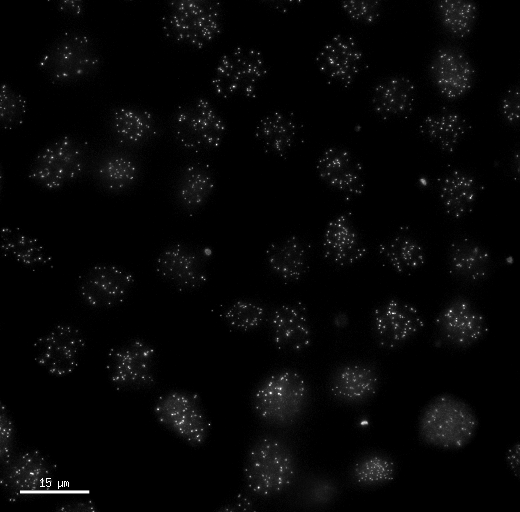

Supplement: Supplementary file 12 — Source data Fig. 7 [file 44318_2024_93_MOESM12_ESM.zip › Figure 7/7B/4A/CENPA.tif]

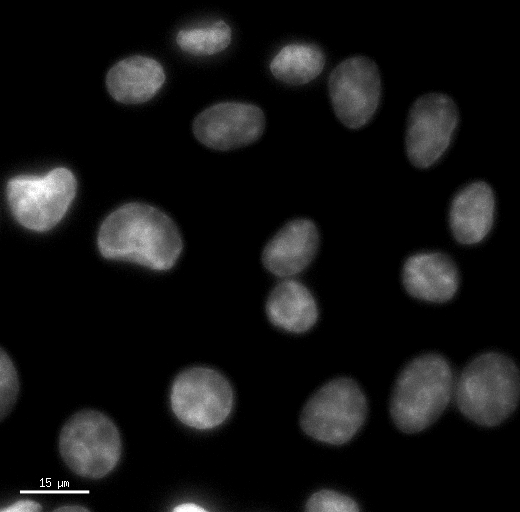

Supplement: Supplementary file 12 — Source data Fig. 7 [file 44318_2024_93_MOESM12_ESM.zip › Figure 7/7B/WT/DAPI.tif]

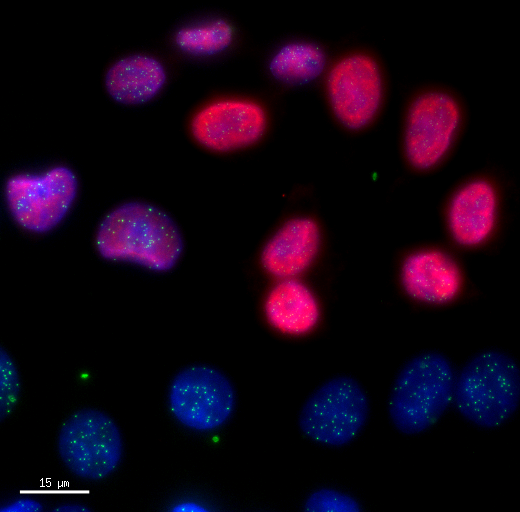

Supplement: Supplementary file 12 — Source data Fig. 7 [file 44318_2024_93_MOESM12_ESM.zip › Figure 7/7B/WT/Merged.tif]

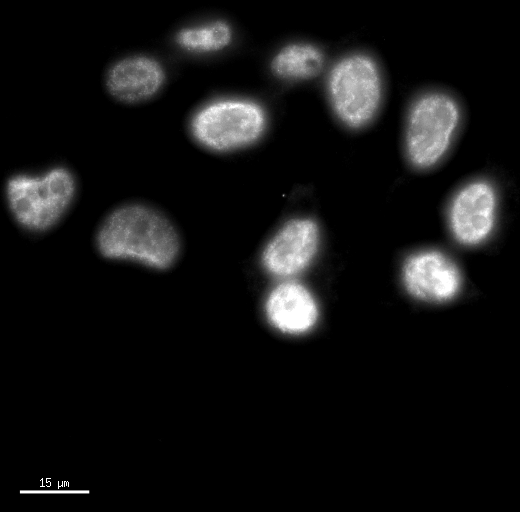

Supplement: Supplementary file 12 — Source data Fig. 7 [file 44318_2024_93_MOESM12_ESM.zip › Figure 7/7B/WT/FLAG-DNAJC9.tif]

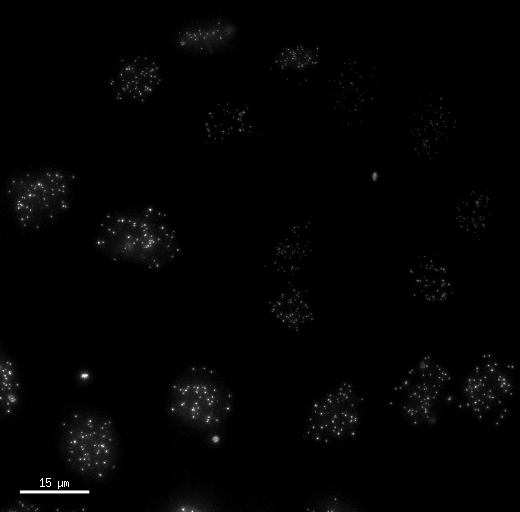

Supplement: Supplementary file 12 — Source data Fig. 7 [file 44318_2024_93_MOESM12_ESM.zip › Figure 7/7B/WT/CENPA.tif]

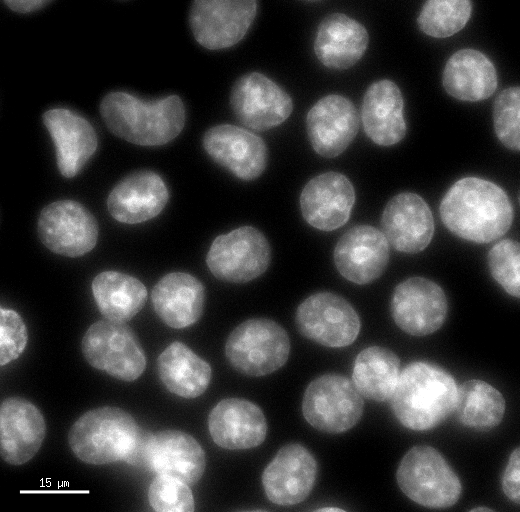

Supplement: Supplementary file 12 — Source data Fig. 7 [file 44318_2024_93_MOESM12_ESM.zip › Figure 7/7B/4AJ/DAPI.tif]

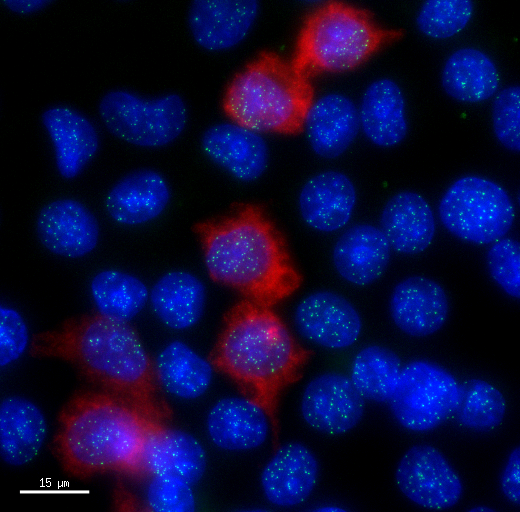

Supplement: Supplementary file 12 — Source data Fig. 7 [file 44318_2024_93_MOESM12_ESM.zip › Figure 7/7B/4AJ/Merged.tif]

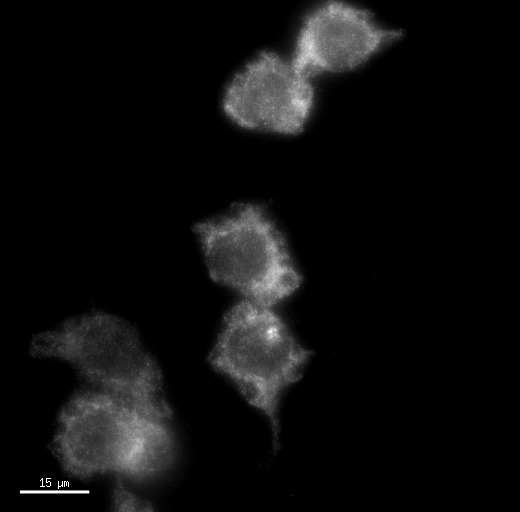

Supplement: Supplementary file 12 — Source data Fig. 7 [file 44318_2024_93_MOESM12_ESM.zip › Figure 7/7B/4AJ/FLAG-DNAJC9.tif]

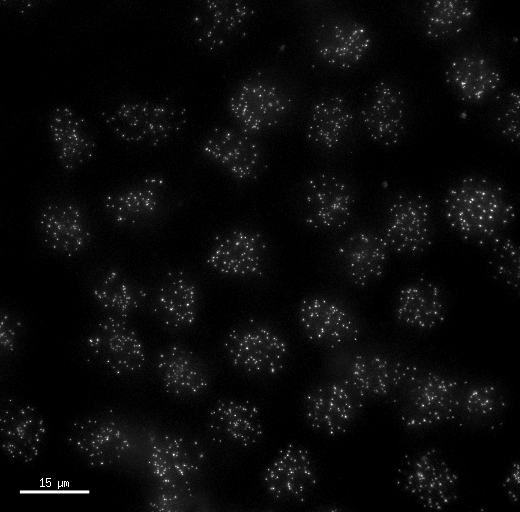

Supplement: Supplementary file 12 — Source data Fig. 7 [file 44318_2024_93_MOESM12_ESM.zip › Figure 7/7B/4AJ/CENPA.tif]
