# Supplementary material for: DNAJC9 prevents CENP-A mislocalization and chromosomal instability by maintaining the fidelity of histone supply chains
Source: EMBO J. 2024 Apr 10;43(11):5. doi: 10.1038/s44318-024-00093-6 (PMC11148058; doi:10.1038/s44318-024-00093-6)
Supplement: Supplementary file 15 — Expanded View Figures [file 44318_2024_93_MOESM15_ESM.pdf]

Expanded View Figures

Figure EV1. DNAJC9 depletion contributes to mislocalization of CENP-A in HeLa parental and HeLa YFP-CENP-A^{High} cells.

(**A**) Exogenous YFP-CENP-A is stably localized to noncentromeric regions in DNAJC9-depleted HeLa YFP-CENP-A^{Low} cells. Representative images of metaphase chromosome spreads prepared from cells extracted with 0.5 M NaCl prior to fixation. Immunostaining was done using anti-GFP and anti-CENP-C antibodies. Scale bar: 10 µm. Scale bar for insets: 1 µm. (**B**) Scatter plots showing YFP-CENP-A intensities corrected for background at the centromeric or noncentromeric regions as described in A, from three biological replicates. Median values are shown above graph. Each circle represents the value from an individual chromosome. Chrs refers to the total number of chromosomes measured per condition from three biological replicates. Mean with standard deviation is shown across all measurements from three biological replicates. *P* values were calculated using Mann–Whitney *U* test. (**C**) CENP-A is mislocalized in DNAJC9-depleted parental HeLa cells. Representative images of metaphase chromosome spreads immunostained for CENP-A in siNeg or siDNAJC9.3-transfected parental HeLa cells. Scale bar: 5 µm. Scale bar for insets: 1 µm. (**D**) Scatter plots showing CENP-A intensities corrected for background at the centromeric or noncentromeric regions as described in (**C**), from three biological replicates. Median values are shown above graph. Each circle represents the value from an individual chromosome. Chrs refers to the total number of chromosomes measured per condition from three biological replicates. Mean with standard deviation is shown across all measurements from three biological replicates. *P* values were calculated using Mann–Whitney *U* test. (**E**) Incidence of CIN phenotypes are not altered in parental HeLa cells with DNAJC9 depletion. Bar graphs showing incidence of micronuclei and defective chromosome segregation in HeLa parental cells transfected with control (siNeg) or siDNAJC9.3. Mean with SD was plotted from three biological replicates and *P* value was calculated using unpaired *t* test. *N* denotes the total number of cells analyzed per condition. (**F**) CENP-A is mislocalized in DNAJC9-depleted HeLa YFP-CENP-A^{High} cells. Representative images of metaphase chromosome spreads immunostained for CENP-A in siNeg or siDNAJC9.3-transfected HeLa YFP-CENP-A^{High} cells. Scale bar: 15 µm. Scale bar for insets: 5 µm. (**G**) Scatter plots showing CENP-A intensities corrected for background at the centromeric or noncentromeric regions as described in (**F**), from three biological replicates. Median intensity is shown above graph. Each circle represents the value from an individual chromosome. Chrs refers to the total number of chromosomes measured per condition from three biological replicates. Mean with standard deviation is shown across all measurements from three biological replicates. *P* values were calculated using Mann–Whitney *U* test. (**H**) Increased incidence of CIN phenotypes in HeLa YFP-CENP-A^{High} cells. Representative IF images showing defects in chromosome segregation in DNAJC9-depleted HeLa YFP-CENP-A^{High} cells immunostained for DNAJC9 and CENP-A. Scale bar: 5 µm. Bar graph shows percent cells with defective chromosome segregation in cells transfected with control (siNeg) or siDNAJC9.3. Mean with SD was plotted from three biological replicates and *P* value was calculated using unpaired *t* test. *N* denotes the total number of cells analyzed per condition.

This is a U.S. Government work and not under copyright protection in the US; foreign copyright protection may apply

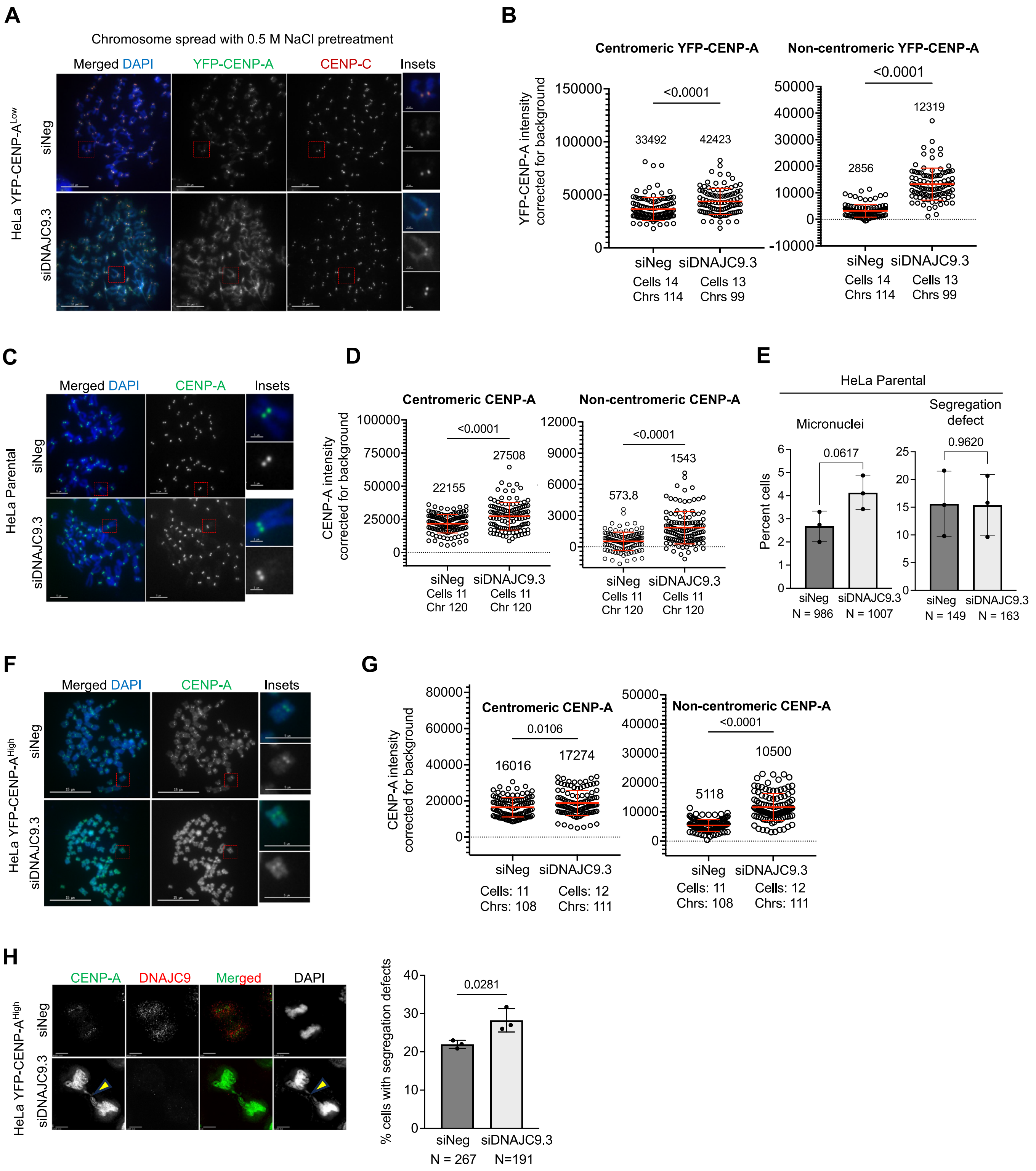

 This is a U.S. Government work and not under copyright protection in the US; foreign copyright protection may apply

A

MS Analysis Workflow

(1) Raw data (3871 proteins - soluble and chromatin fractions)

(2) Filtering reverse hits, only identified by site and contaminants (3713 proteins), the second replicate for the siDNAJC9 condition was omitted due to lack of knockdown.

(3) Filtering for observations in 100 % of experiments siCTRL or siDNAJC9 or siCAF1B in soluble CENPA IP-MS replicates (soluble: 1813 proteins)

(4) Filtering for enrichment over beads in any condition (955 proteins, including 261 imputed ratios identified in ≤1 replicate in the bead only condition) - panel B

(5) Bait normalisation - panel C

(6) siCTRL vs siDNAJC9 and siCTRL vs siCAF1B volcano plots - no imputed ratios - panels D & E

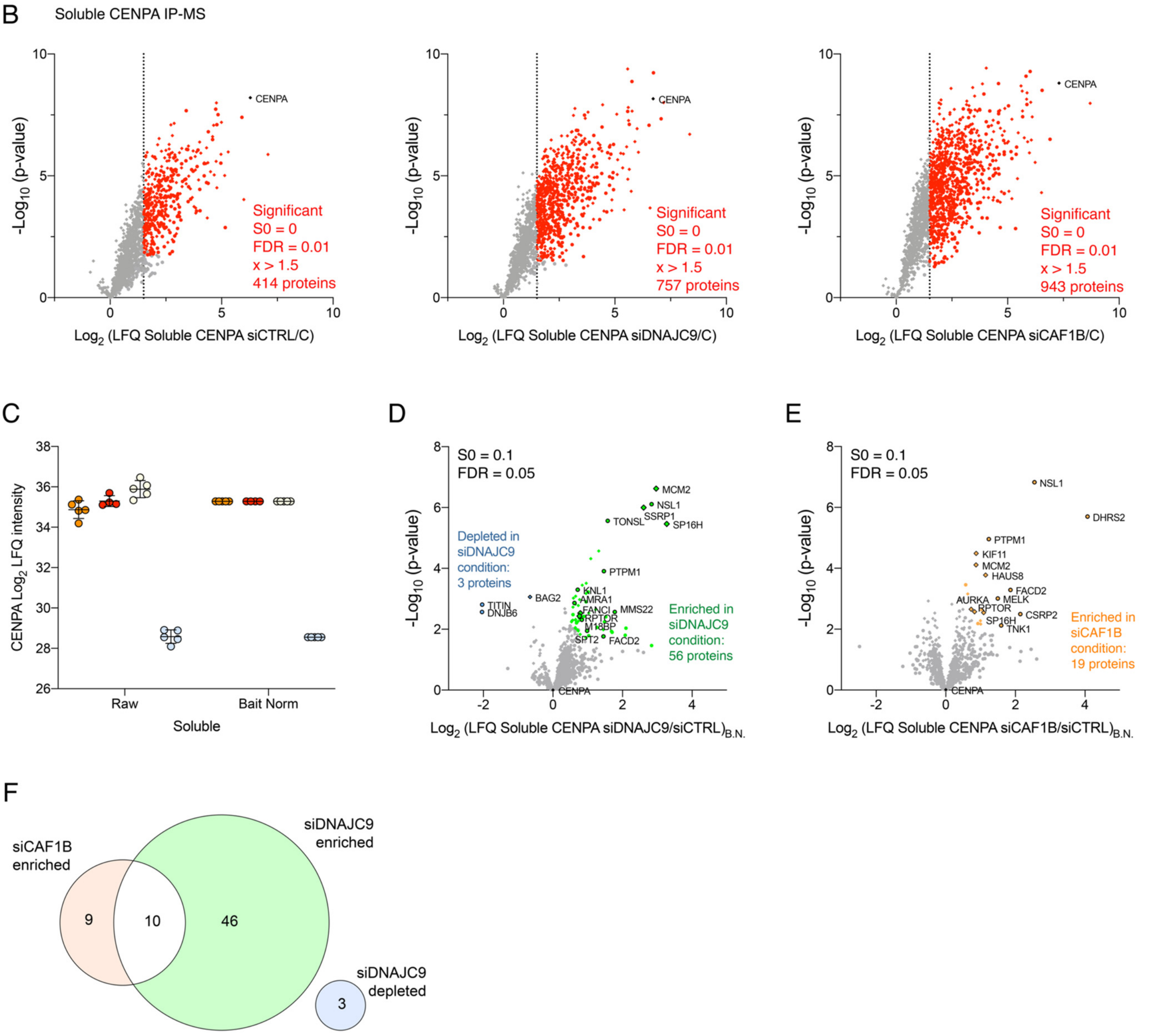

Figure EV2. Soluble CENP-A-Flag-HA IP-MS data analysis.

(**A**) Overview of data analysis workflow. Data from $n = 5$ biological replicates and $n = 4$ biological replicates for siDNAJC9 conditions. See also Dataset EV4. (**B**) Volcano plots showing enrichment of proteins associated with soluble CENP-A-Flag-HA in siCTRL, siDNAJC9 and siCAF1B conditions compared to "C" uninduced control conditions. Factors highlighted in red are significant based on two-sided *T* tests with $S0 = 0$, $FDR = 0.01$ and have a minimum Log2 fold change of 1.5 in their ratio of LFQ intensity (siRNA/C). Circular data points represent factors where ratios were imputed due to a lack of sufficient observations (>1) in uninduced control condition biological replicates. Proteins referred to by human UniProt protein identification code. (**C**) CENP-A LFQ intensity in purifications before and after bait normalization. (**D**, **E**) Volcano plots showing changes in bait normalized LFQ intensities ($LFQ_{B.N.}$) for factors specifically enriched with soluble CENP-A-Flag-HA comparing intensity changes in (**D**) siDNAJC9 and (**E**) siCAF1B conditions compared to DOX+ siCTRL conditions. Colors were used to highlight CENP-A and factors that show significant changes as assessed by two-sided *T* tests with $S0 = 0.1$ and $FDR = 0.05$. Proteins referred to by human UniProt protein identification code. (**F**) Venn diagram color-coded as in (**D**, **E**), showing the overlap of factors classified as statistically changing in siDNAJC9 and siCAF1B conditions calculated using InteractiVenn (Heberle et al, 2015).

This is a U.S. Government work and not under copyright protection in the US; foreign copyright protection may apply

A

MS Analysis Workflow

(1) Raw data (3871 proteins - soluble and chromatin fractions)

(2) Filtering reverse hits, only identified by site and contaminants (3713 proteins), the second replicate for the siDNAJC9 condition was omitted due to lack of knockdown.

(3) Filtering for observations in 100 % of experiments siCTRL or siDNAJC9 or siCAF1B in chromatin CENPA IP-MS replicates (1395 proteins)

(4) Filtering for enrichment over beads in any condition (1045 proteins, including 342 imputed ratios identified in ≤1 replicate in the bead only condition) - panel B

(5) Bait normalisation - panel C

(6) siCTRL vs siDNAJC9 and siCTRL vs siCAF1B volcano plots - no imputed ratios - panels D & E

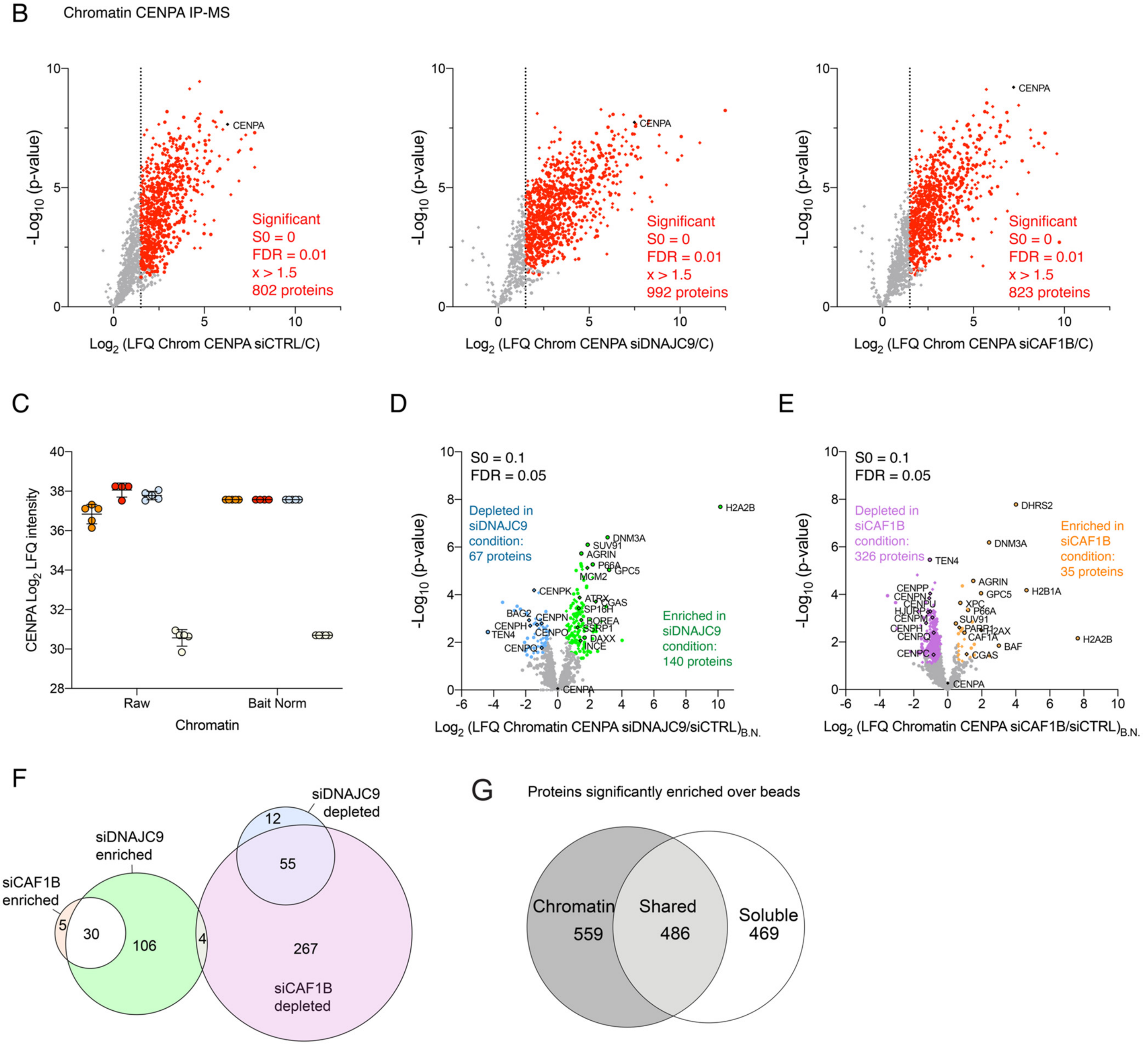

Figure EV3. Chromatin CENP-A-Flag-HA IP-MS data analysis.

(**A**) Overview of data analysis workflow. Data from $n = 5$ biological replicates and $n = 4$ biological replicates for siDNAJC9 conditions. See also Dataset EV4. (**B**) Volcano plots showing enrichment of proteins associated with chromatin-bound CENP-A-Flag-HA in siCTRL, siDNAJC9 and siCAF1B conditions compared to "C" uninduced control conditions. Factors highlighted in red are significant based on two-sided *T* tests with S0 = 0, FDR = 0.01 and have a minimum Log2 fold change of 1.5 in their ratio of LFQ intensity (siRNA/C). Circular data points represent factors where ratios were imputed due to a lack of sufficient observations (>1) in uninduced control condition replicates. Proteins referred to by human UniProt protein identification code. (**C**) CENP-A LFQ intensity in purifications before and after bait normalization. (**D**, **E**) Volcano plots showing changes in bait normalized LFQ intensities ($LFQ_{B.N.}$) for factors specifically enriched with chromatin-bound CENP-A-Flag-HA comparing intensity changes in (**D**) siDNAJC9 and (**E**) siCAF1B conditions compared to DOX+ siCTRL conditions. Colors were used to highlight CENP-A and factors that show significant changes as assessed by two-sided *T* tests with S0 = 0.1 and FDR = 0.05. Proteins referred to by human UniProt protein identification code. (**F**) Venn diagram, color-coded as in (**D**, **E**), showing the overlap of factors classified as statistically changing in siDNAJC9 and siCAF1B conditions calculated using InteractiVenn (Heberle et al, 2015). (**G**) Venn diagram, showing the overlap of factors specifically enriched with soluble and chromatin-bound CENP-A-Flag-HA calculated using InteractiVenn (Heberle et al, 2015).

This is a U.S. Government work and not under copyright protection in the US; foreign copyright protection may apply

A

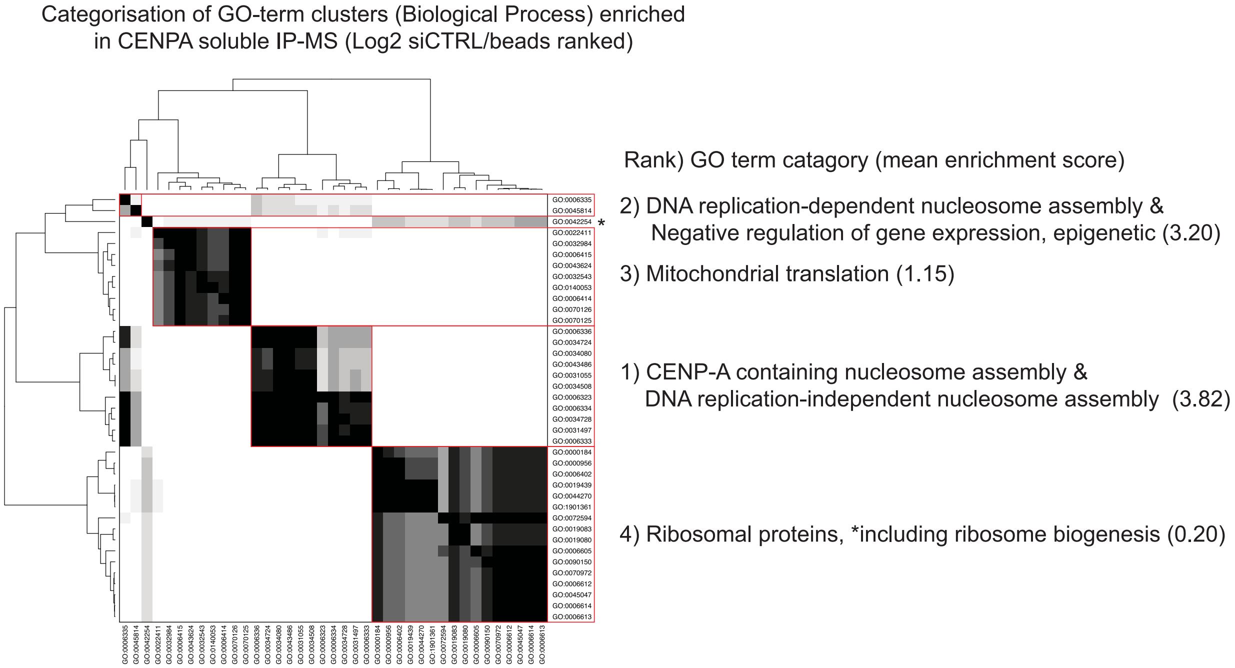

B

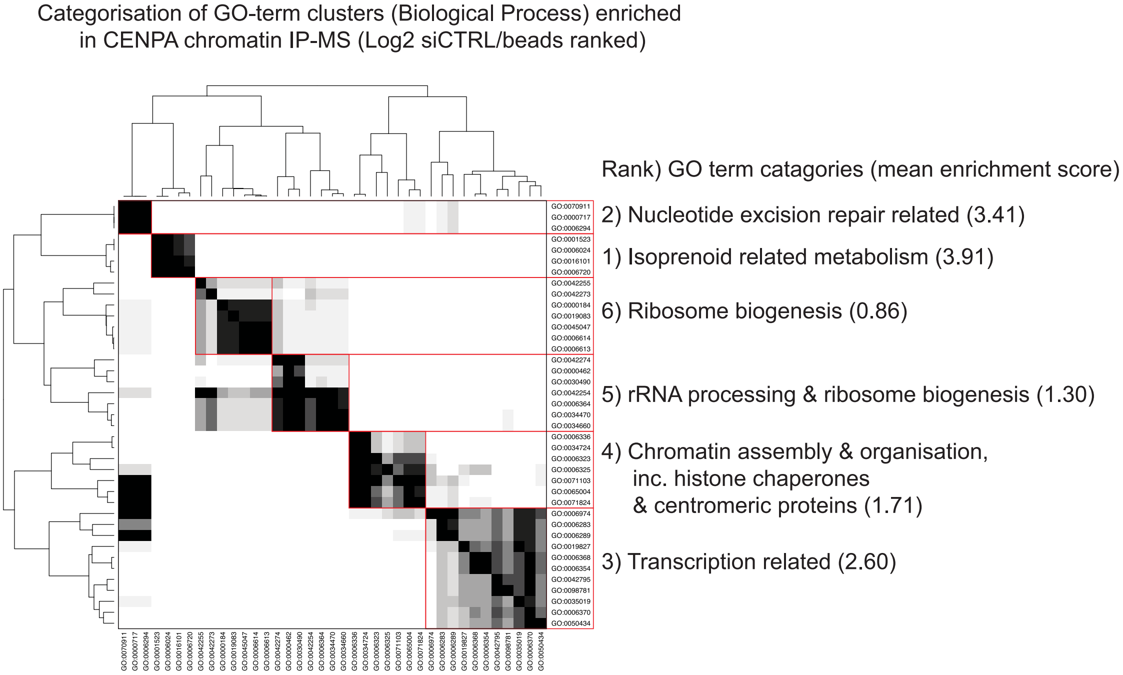

C

GO terms representing highest enriched clusters:

Soluble CENPA IP-MS enriched (count, enrichment, FDR)
GO:0034080, CENP-A containing nucleosome assembly (13, 5.28, 7.34E-06)
GO:0006335, DNA replication-dependent nucleosome assembly (6, 4.36, 0.0007)
Chromatin CENPA IP-MS enriched (count, enrichment, FDR)
GO:0006720, Isoprenoid metabolic process, (8, 3.64, 0.0054)
GO:0070911, Global genome nucleotide-excision repair (9, 3.41, 0.0054)
GO:0006354, DNA-templated transcription, elongation (24, 2.51, 0.00082)
GO:0006336, DNA replication-independent nucleosome assembly (26, 2.32, 0.0075)

This is a U.S. Government work and not under copyright protection in the US; foreign copyright protection may apply

Figure EV4. Biological processes enriched with soluble and chromatin-bound CENP-A.

(**A**, **B**) GO-analysis of proteins ranked by enrichment over beads in (**A**) soluble and (**B**) chromatin CENP-A IP-MS experiments were input into the String-db "Proteins with Values/Ranks - Functional Enrichment Analysis". To assess the redundancy of Biological Process GO-terms enriched, terms were clustered by the percentage identity of their associated proteins. GO-term clusters of terms were ranked by the mean fold enrichment of associated GO-terms in the String-db analysis. See also Dataset EV4. (**C**) GO-terms representing most highly enriched GO-term clusters defined in A and B.

This is a U.S. Government work and not under copyright protection in the US; foreign copyright protection may apply

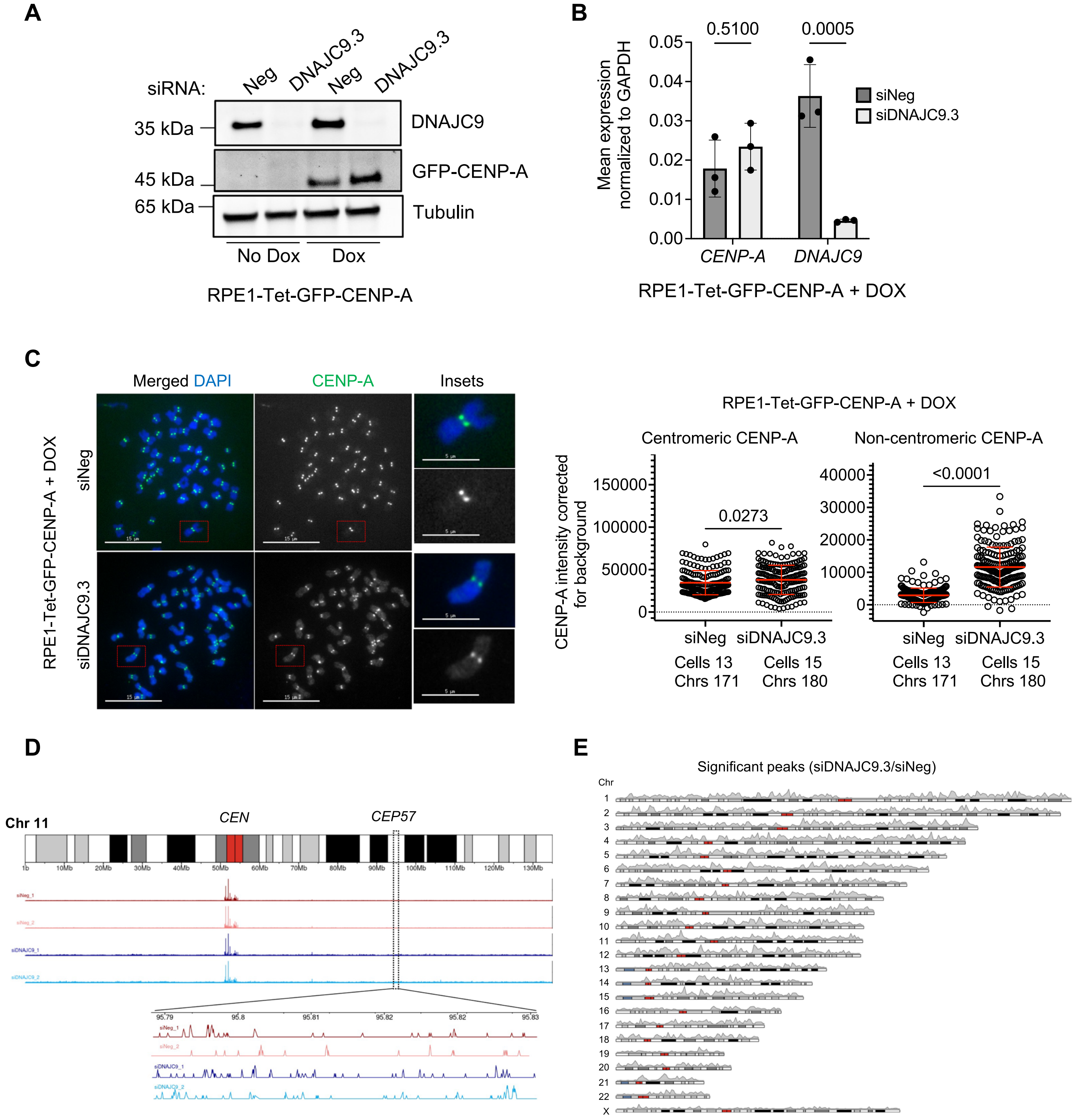
A
siRNA:
Neg
DNAJC9.3
Neg
DNAJC9.3
35 kDa
DNAJC9
45 kDa
GFP-CENP-A
65 kDa
Tubulin
No Dox
Dox
RPE1-Tet-GFP-CENP-A
B
0.5100
0.0005
Mean expression normalized to GAPDH
siNeg
siDNAJC9.3
CENP-A
DNAJC9
RPE1-Tet-GFP-CENP-A + DOX
C
Merged DAPI
CENP-A
Insets
RPE1-Tet-GFP-CENP-A + DOX
siNeg
siDNAJC9.3
RPE1-Tet-GFP-CENP-A + DOX
Centromeric CENP-A
Non-centromeric CENP-A
CENP-A intensity corrected for background
0.0273
<0.0001
siNeg
siDNAJC9.3
Cells 13
Chrs 171
Cells 15
Chrs 180
D
Chr 11
CEN
CEP57
E
Significant peaks (siDNAJC9.3/siNeg)
Chr

Figure EV5. DNAJC9 depletion promotes CENP-A mislocalization in RPE1-Tet-GFP-CENP-A cells without affecting CENP-A RNA levels.

(**A**) Western blot of whole-cell extracts showing DNAJC9 depletion in siNeg or siDNAJC9.3-transfected RPE1-Tet-GFP-CENP-A cells with or without 1 µg/ml DOX induction for 48 h post-siRNA transfection. Alpha-tubulin was used as the loading control. (**B**) Transcript levels of *CENP-A* are not altered in DNAJC9-depleted RPE1-Tet-GFP-CENP-A cells treated with 1 µg/ml DOX for 48 h. Bar graphs showing mean RNA levels of *CENP-A* and *DNAJC9* normalized to *GAPDH* in control and DNAJC9-depleted cells using RT-qPCR. Mean values with standard deviation from three biological replicates were plotted and p values were calculated from two-way ANOVA with Sidak's multiple correction test. (**C**). CENP-A is mislocalized in DOX-treated RPE1-Tet-GFP-CENP-A cells with DNAJC9 depletion. (Left panel) Representative images of metaphase chromosome spreads immunostained for CENP-A in siNeg or siDNAJC9.3-transfected RPE1-Tet-GFP-CENP-A cells treated with 1 µg/ml DOX for 48 h post-siRNA transfection. Scale bar: 15 µm. Scale bar for insets: 5 µm. (Right) Scatter plots showing CENP-A intensities corrected for background at the centromeric or noncentromeric regions as described above, from three biological replicates. Each circle represents the value from an individual chromosome. Chrs refers to the total number of chromosomes measured per condition from three biological replicates. Mean with standard deviation is shown across all measurements from three biological replicates. *P* values were calculated using Mann–Whitney *U* test. (**D**) Example of binding profile of CENP-A on chromosome 11 in the CENP-A CUT&RUN sequencing datasets with siRNA treatments indicated. Peaks spanning the centromeric region (*CEN*) and an adjacent representative noncentromeric region (*CEP57*) are highlighted. Magnified view of noncentromeric localization of CENP-A at *CEP57* is also shown. (**E**) Karyoplot showing the distribution of the 17,341 significantly enriched (adjusted *P* value less than or equal to 0.05) CENP-A CUT&RUN peaks identified in DNAJC9-depleted GFP-CENP-A expressing RPE1 cells compared to siNeg-treated control. Plot represents the average from two biological replicates.

This is a U.S. Government work and not under copyright protection in the US; foreign copyright protection may apply
